# The effect of chronic low back pain on postural control during quiet standing: A meta-analysis

**DOI:** 10.1038/s41598-023-34692-w

**Published:** 2023-05-16

**Authors:** Jinhan Park, Vinh Q. Nguyen, Rachel L. M. Ho, Stephen A. Coombes

**Affiliations:** 1 Laboratory for Rehabilitation Neuroscience, Department of Applied Physiology and Kinesiology, University of Florida, PO Box 118206, Gainesville, FL 32611 USA; 2 Department of Biomedical Engineering, University of Florida, Gainesville, FL 32611 USA

**Keywords:** Chronic pain, Disability

## Abstract

Low back pain (LBP) has been associated with altered body sway during quiet standing, but the pattern of results is inconsistent. The purpose of this meta-analysis is to examine the effects of vision (eyes open, eyes closed) and changing the support surface (foam surface, firm surface) on postural sway during quiet standing in individuals with chronic LBP (cLBP). Five electronic databases were searched on March 27th, 2022. Of 2,856, 16 studies (n = 663) were included. Across all conditions, we found a positive and medium effect size (g = 0.77 [0.50, 1.04]) that represented greater body sway in individuals with cLBP. Subgroup analyses revealed medium effects during eyes open conditions (firm surface: g = 0.60 [0.33, 0.87]; foam surface: g = 0.68 [0.38, 0.97]), and large effects during eyes closed conditions (firm surface: g = 0.97 [0.60, 1.35]; foam surface: g = 0.89 [0.28, 1.51]). We quantified effects of self-reported pain and found a moderate effect during eyes closed plus firm surface conditions (Q = 3.28; *p* = 0.070). We conclude that cLBP is associated with increased postural sway, with largest effect sizes evident when vision is removed and when self-reported pain intensity is higher.

## Introduction

Low back pain (LBP) is a major public health concern, affecting up to 85% of adults at some point throughout their lifetime^1^. Although many individuals recover from an acute episode of low back pain, a subset of individuals go on to develop chronic low back pain (cLBP), which is pain lasting for 12 or more weeks^2^. cLBP leads to increases in disability in normal activity and limitation in the workplace^3^. The cost of cLBP is estimated to range from $259 million to $71.6 billion per year based on healthcare costs and missed work days^4^. A major concern for individuals with cLBP is instability in body movements during quiet standing^5–7^ which directly relates to the risk of falling^8,9^.

Stable balance utilizes sensory input from visual, vestibular, and proprioceptive systems^10^. During quiet standing, pain-free individuals rely on proprioceptive information not only from the lumbar muscles but also from other postural muscles including those that control the ankle. Individuals with low back pain will often restrict trunk movement in an effort to reduce low back pain^7^. Over time, this change in postural control reduces reliance on lumbar proprioception^5,7^, leading to an increase in motion perception threshold^11^. In the long term, this alteration leads to greater dependence on other sensory information to compensate for the loss of input from lumbar proprioception^12^. A consequence of this change in the weighting of sensory input becomes most apparent when individuals must control movements in an unpredictable environment. A recent systematic review by Koch and Hänsel^5^ reported that cLBP is associated with more restricted trunk movement when sensory input is manipulated by altering the stability of the surface or the presence of visual feedback.

One of the most common analytical methods to evaluate postural control is to calculate center of pressure (CoP) excursions during quiet standing. CoP is measured using a force plate and represents the change in the center of mass in the sagittal and frontal planes. Both the velocity of the change and the magnitude of the change can be calculated. There is a growing body of evidence linking cLBP with greater and faster CoP movements during quiet standing on stable surfaces with vision and with no-vision^6,13^, as well as when standing on an unstable surface with vision^14,15^ and without vision^13^. This is consistent with several systematic reviews^5,16–18^, which suggest that LBP leads to a shift in postural control from the lumbar spine to the ankles, and controlling posture at the ankles increases magnitude of sway^19^. However, other studies have reported smaller and slower CoP movements during quiet standing with vision^20,21^ and no-vision^20^. Several studies have also reported no difference in CoP between individuals with and without cLBP^22–24^. Differences in task conditions, pain levels, and CoP calculations may account for ambiguity across studies. A meta-analytic approach offers one solution to resolve findings across studies.

The purpose of the current study is to combine and statistically compare results from studies on cLBP and postural control. First, we compared overall effect sizes of velocity and magnitude of CoP sway between individuals with and without cLBP. Second level meta-analyses were conducted by statistically comparing groups within four conditions that varied as a function of proprioceptive input (Firm vs. Foam surfaces) and visual input (eyes open vs. closed). Finally, we analyzed the effects of movement direction and self-reported pain intensity as moderator variables. We hypothesized that people with cLBP would have greater and faster CoP movement and that effect sizes would be larger when proprioceptive and visual input is reduced.

## Methods

This meta-analysis was conducted by following the guidelines of the PRISMA checklist^25^ (see Table S1 in Supplementary materials 1).

### Search strategy

The following five databases were used, PubMed, MEDLINE, CINAHL, SPORTDiscus, and APA PsycArticles, to search for articles published from 1979 to 27th March 2022, using the keywords with parentheses, Boolean operators, and field codes. We used the keywords: (low back pain) and (postural* or stability or balance or postural sway or postural balance or postural stability or CoP or center of pressure) and not (meta* or systematic review or review). The full keywords can be found in Supplementary materials 1. Articles not written in the English language were excluded using the filtering function in each database. To find published articles, JP, who is the first author on the paper, conducted the search twice: the first search was conducted on 1st November 2021 and the second on 27th March 2022.

### Study selection

The procedure to select studies was conducted using the following steps. First, duplicate studies were removed using EndNote X9 (Clarivate)^26^. According to the inclusion and exclusion criteria, a researcher (JP) screened titles and abstracts of all studies, and then two researchers (JP and VN) independently rescreened the studies through the full-text versions. In case of disagreement, the last researcher (SAC) was consulted for the final decision. The final studies were selected in our meta-analysis. We included studies that: (1) recruited cLBP subjects with pain duration of greater than or equal to 3 months, (2) recruited adult population ranged from 18 to 65 years, (3) compared a cLBP group and healthy control group (HC), (4) used cross-sectional design, and (5) used CoP parameters during bipedal quiet standing. Exclusion criteria were studies that: (1) recruited athletes with cLBP or cLBP subjects who have had surgery for lumbar disc herniation or other diseases, (2) recruited recurrent or episodic LBP subjects who reported semicontinuous LBP or at least one episode of LBP during the previous 3 months, (3) did not include a control group, (4) did not measure CoP variables (e.g., the center of mass, the center of gravity, and the joint angles) to compare postural control between groups, (5) did not investigate postural control during a bipedal quiet standing task (i.e., perturbation tasks, balance-dexterity tasks, anticipation postural control task, one leg standing task, and dual tasks are exclusion criteria), and (6) did not report statistical results for calculating the effect size.

### Data extraction

To calculate the pooled effect size, all the CoP outcomes were extracted from included studies. CoP data was classified according to body sway and body sway velocity to reduce the heterogeneity. Body sway included the sway amplitude, displacement, area, and dispersion, while body sway velocity variables represent the velocity of the CoP^18^. Both parameters were collected as means with their standard deviation and sample size. If a study reported a median and interquartile range, those values were converted into a mean and standard deviation based on the Quantile estimation method^27^ to calculate an effect size. In the case that a study did not report group level descriptive data, effect sizes were calculated using a combination of sample size, mean differences, p-values, and t-values. If a study reported a standard error, the standard error was converted into a standard deviation based on the corresponding sample size. Furthermore, we extracted demographic information (i.e., sex, age, height, and weight) and pain related characteristics when available (i.e., pain intensity, pain duration, and disability). Two authors, JP and VN, extracted and checked data.

### Effect size

Hedges’ g was used to calculate effect sizes of each studies’ outcome. The rationale for using Hedges’ g is that it is less affected by the impact of sample size compared to Cohens’ d^28^. The Hedges’ g is interpreted based on 3 stages: (1) values from 0.2 to 0.49 represent a small effect size; (2) values from 0.5 to 0.79 represent a medium effect size; (3) values greater than or equal to 0.8 represent a large effect size.

### Meta-analyses

We first calculated overall effect sizes for group differences in body sway and body sway velocity during quiet standing across all experimental conditions (i.e., vision and proprioception). Next, we calculated effect sizes for group differences in body sway and body sway velocity across different experimental conditions: (1) on a firm surface with eyes open (Firm with EO), (2) a firm surface with eyes closed (Firm with EC), (3) on a foam surface with eyes open (Foam with EO) and (4) on a foam surface with eyes closed (Foam with EC). In each experimental condition we also assessed moderator variables based on direction of body sway and body sway velocity (anterior–posterior [AP], the medial–lateral [ML], and both [AP-ML] directions) and pain (low and high). For the levels of pain intensity, the results from a visual analogue scale (VAS) or numerical rating scale (NRS) were extracted. In the case where an included study reported pain scores based on the range from 0 to 100, their scores were converted to a value from 0 to 10. Pain intensity was then classified into two levels using the median value across all studies (4.73): a higher level of LBP is equal to and more than the median, which we labeled ‘High’, whereas a lower level of LBP is less than the median and is labeled as ‘Low’.

### Quality assessment

The quality assessment tool (QAT) for observational cohort and cross-sectional studies^29^ was used to evaluate the quality of each study included in the meta-analysis. The QAT contains fourteen questions to access internal validity. The details on the questions are described in the supplementary material (Table S3). All the questions were rated as being positive (“Yes”), negative (“No”), cannot determine (“CD”), not applicable (“AP”), or not reported (“NR”). Two authors, named as JP and VN, rated the quality of studies. The disagreements were discussed until consensus was reached. In cases where JP and VN could not reach an agreement, they discussed it with SAC. The quality of every study was evaluated by “Good”, “Fair”, and “Poor.”

### Data analysis

The Comprehensive Meta-Analysis software package version 3 (CMA; BioStat, Englewood, New Jersey) was used to calculate effect sizes and 95% confidence intervals using a random effect model for each analysis. If a study included in the meta-analysis had multiple CoP outcomes, we calculated multiple effect sizes corresponding to CoP outcomes and then averaged those effect sizes into a single effect size per study according to body sway or body sway velocity and standing conditions. For instance, Caffaro et al.^13^ reported 12 different CoP outcomes of body sway that were different according to experimental conditions and directions (See the Supplementary material 2). When the meta-analysis for body sway was conducted, the CMA program calculated 12 effect sizes from 12 CoP results and then averaged all effect sizes into a single effect, which was 0.77 (see the first row in Fig. 2a). An I^2^ statistic and prediction interval were used to assess the heterogeneity for each analysis. I^2^ values higher than 75% represent high heterogeneity, and the prediction interval (PI) represents the range of true effect sizes for 95% of all comparable studies^30^. To assess publication biases for each analysis, Egger’s regression test was first used^31^. The significant level for the bias was set to 0.05. If publication bias was found through Egger’s regression test, Duval and Tweedie’s Trim and Fill^32^ was conducted to reassess publication bias using a funnel plot which can test the symmetry of effect sizes of included studies. Asymmetric funnel plots suggest that publication bias may exist. Using the trim and fill approach, some data was imputed to make the figure look symmetric, and then the changed overall effect size including the imputed data was compared to the previous overall effect size without imputed data. Unless 95% confidence intervals of both overall effect sizes were overlapped, there is an effect of publication bias. For subgroup analyses, Q-statistics were used to assess the moderator effects of movement direction (AP, ML, and AP-ML) and pain intensity (Low and High). The significant level for Q-statistics was set to 0.10. Effect sizes for sublevels of variables (e.g., AP, ML, AP-ML, Low, and High) were statistically analyzed. Significant *p*-values were set at a level of 0.05, corrected using the false discovery rate (FDR). Meta- or subgroup analyses with only one study were excluded^33^.

### GRADE assessment

Grading of recommendations, assessment, development, and evaluations (GRADE) was used to evaluate the evidence certainty based on five subcategories: ‘Risk of bias’, ‘Inconsistency’, ‘Indirectness’, ‘Imprecision’, and ‘Publication bias’. The ratings for those categories were ‘No serious’, ‘Serious’, and ‘Very serious’. The level of evidence certainty was downgraded if the meta-analysis (i) included 25% of studies with poor quality assessed by QAT (1 level of Risk of bias) or all studies with poor quality (2 level of Risk of bias), (ii) showed higher than 50% of I^2^ (1 level of Inconsistency)—I^2^ values have been used previously to evaluate inconsistency in CoP outcomes^34,35^—(iii) did not include direct evidence related to the main question (1 level of Indirectness), (vi) included less than the optimal sample size (n = 244) calculated based on 5% of margin of error, 95% confidence intervals, and total sample size (n = 663; 1 level of Imprecision), and (v) showed publication bias resulting from Egger’s regression and trim and fill test (1 level of Publication bias). Combining all the ratings, the certainty of the evidence in meta-analyses were interpreted based on four levels: ‘High’, ‘Moderate’, ‘Low’, and ‘Very low certainty’. The high certainty of evidence represents high confidence that the true effect is close to the effect of the estimate, while very low certainty represents very little confidence that the true effect is close to the effect of the estimate. Initial ratings began at a low level since all studies included in the meta-analyses were observational studies. However, the level can be graded up (i) if the pooled Hedges’ g was higher than or equal to 0.8, (ii) evidence of a dose–response relation was found, and (iii) if all the bias and/or confounds were plausible^36,37^.

## Results

**Figure 1:**
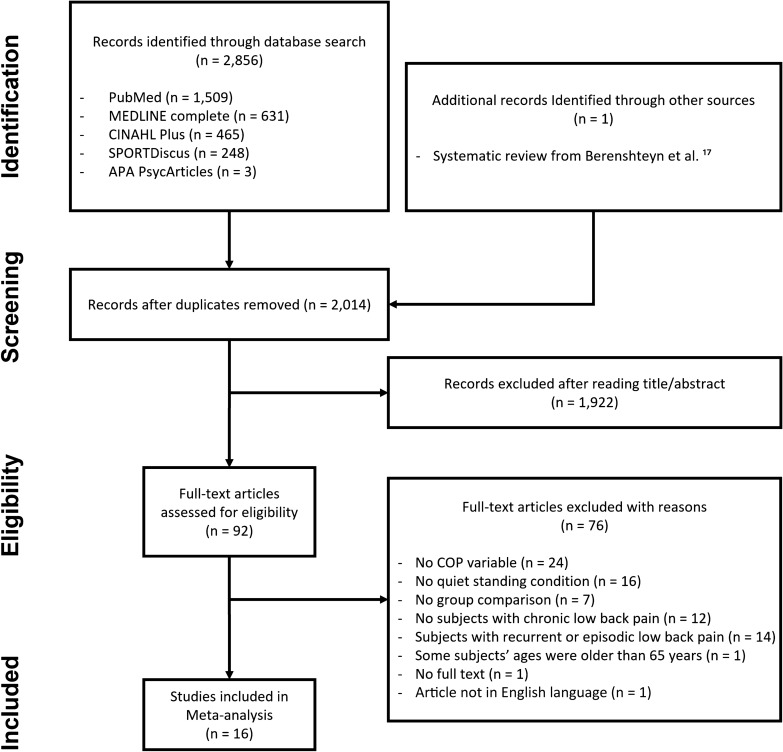
*PRISMA flow chart. Note*. This figure represents the results from the second search using PubMed, MEDLINE, CINAHL plus, SPORTDiscus, and APA PsycArticles.

Fig. S1 in supplementary material 1 shows the PRISMA flow chart for the first search. Figure 1 shows the PRISMA flow chart for the second and final search. The search strategies initially yielded 2856 relevant articles, and one additional article^38^ extracted from a systematic review^17^. Among them, 842 duplicates were removed, leaving a total of 2014 articles. After investigating titles and abstracts, 1922 articles were excluded. From the remaining 92 articles, we excluded an additional 76 articles based on the criteria outlined in Fig. 1. Additional information on why studies were excluded from the meta-analyses is shown in Table S2. A total of 16 articles were included in this meta-analysis.

### Demographic data

Table 1 represents demographic information for cLBP and HC groups for each article. The bottom three rows in the Total section of Table 1 indicate the summed and averaged demographic information on the overall population of cLBP, HC, and total individuals respectively. The first row in the Total section of Table 1 shows demographic information on cLBP group. Total sample size of the cLBP group was 345 (Female: 153; Male: 98; no information on sex: 94). The average age of cLBP individuals was 35.4 ± 8.2 years. The mean height was 169.3 ± 5.9 cm, and weight was 69.9 ± 8.5 kg. Intensity of low back pain was measured using VAS and NRS. Mean pain intensity was 4.8 ± 1.1 (2.5–6.7). The duration of cLBP ranged from 3 to 139 months. The second row in the Total section of Table 1 represents the demographic information on the HC group. The total healthy sample size was 318 (Female: 115; Male: 63; no information on sex: 140). The mean of age in the HC group was 32.7 ± 6.4 years. The height was 167.8 ± 4.4 cm, and weight was 65.7 ± 6.7 kg. The total pooled population (last row) was 663 (Females: 268; Males: 161; no mention of sex: 234). Average age was 34.1 ± 7.3 years (18–61 years). Average height was 168.6 ± 5.2 cm (160–174.9 cm) and average weight was 67.8 ± 7.8 kg (55.2–77.6 kg).

**Table 1:** Demographic data from studies included in the meta-analysis.

Author	Group	Sample (F/M)	Age (yrs)	Heights (cm)	Weight (kg)	Pain level	Disability	Pain duration
Abbasi et al.^42^	cLBP	20 (10/10)	42.0 ± 5.5	–	–	4.7 ± 1.8 (VAS)	20.9 ± 11.1 (ODI)	> 3 mths
	HC	20 (10/10)	36.0 ± 8.4	–	–	–	–	–
Caffaro et al.^13^	cLBP	21 (10/11)	43.4 ± 7.8	169.0 ± 9.1	73.5 ± 13.9	4.1 ± 1.5 (VAS)	8.0 ± 4.0 (RM)	85.5 ± 17.1 mths
	HC	23 (17/6)	38.8 ± 8.9	165.0 ± 4.8	65.9 ± 11.2	–	–	–
Cana–Pino et al.^14^	cLBP	30	33.7 ± 7.0	171.3 ± 0.1	72.8 ± 9.2	6.7 ± 1.1 (NRS)	9.0 ± 5.0 (RM)	37.9 ± 38.5 mths
	HC	30	30.7 ± 6.1	170.1 ± 0.1	68.6 ± 8.3	–	–	–
da Silva et al.^20^	cLBP	10 (5/5)	34.4 ± 2.9	168.0 ± 8.2	77.6 ± 12.6	4.5 ± 2.2 (VAS)	7.6 ± 5.2 (RM)	51.6 ± 12.0 mths
	HC	10 (5/5)	33.6 ± 2.5	166 ± 9.2	66.5 ± 11.7	–	–	–
della Volpe et al.^23^	cLBP	12 (5/7)	35.4 ± 12.4	174.9 ± 9.2	–	–	7.8 ± 7.2 (ODI)	5.2 ± 3.3 yrs
	HC	12	–	–	–	–	–	–
Hamaoui & Bouisset^40^	cLBP	10 (0/10)	33	181	77	–	–	> 3 mths
	HC	10 (0/10)	31	175	69	–	–	–
Lafond et al.^21^	cLBP	12	41.5 ± 11.7	172.0 ± 10.6	74.6 ± 15.4	2.5 ± 2.4 (VAS)	12.6 ± 7.3 (%: ODI)	> 6 mths
	HC	12	40.0 ± 12.6	167.3 ± 9.8	68.5 ± 15.5	–	–	–
Mann et al.^38^	cLBP	10 (10/0)	20.7 ± 2.1	165.0 ± 4.0	57.6 ± 0.6	6.0 ± 2.0 (VAS)	–	> 3 mths
	HC	10 (10/0)	20.2 ± 1.7	166 ± 3.0	56.7 ± 0.2	–	–	–
Nogueira et al.^6^	cLBP	32	44.0 ± 9.0	166.0 ± 9.0	76.8 ± 17.6	4.2 ± 2.3 (VAS)	–	–
	HC	33	40.0 ± 9.2	169.0 ± 9.0	72.6 ± 15.6	–	–	–
Popa et al.^22^	cLBP	13 (6/7)	35.1 ± 11.9	174.3 ± 9.1	76.5 ± 17.9	–	14.2 ± 14.5 (%: ODI)	5.2 ± 3.3 yrs
	HC	13	32.2 ± 7.2	174.4 ± 7.5	69.5 ± 12.7	–	–	–
Ringheim et al.^43^	cLBP	17 (10/7)	39.0 ± 5.4	177.5 ± 6.5	81.7 ± 15.7	5 ± 1.7	21.1 ± 7.8 (&: ODI)	139 ± 119 mths
	HC	20	40.2 ± 5.4	174.6 ± 8.9	77.5 ± 16.7	–	–	–
Shigaki et al.^24^	cLBP	10 (5/5)	33.0 ± 8.0	170.0 ± 6.0	67.0 ± 9.0	5.0 ± 2.0 (VAS)	8.0 ± 4.0 (RM)	> 3 mths
	HC	10 (5/5)	30.0 ± 4.0	165.0 ± 12.0	64.0 ± 8.0	–	–	–
Sundaram et al.^44^	cLBP	20	21.7 ± 1.8	161.5 ± 14.5	61.0 ± 12.5	4.7 ± 3.5 (VAS)	–	> 3 mths
	HC	20	23.8 ± 2.4	166.0 ± 9.7	57.7 ± 10.7	–	–	–
Wang et al.^39^	cLBP	30 (21/9)	27.5 ± 4.76	165.4 ± 7.13	56.22 ± 9.94	5.39 ± 1.15 (VAS)	7.90 ± 3.81 (ODI)	> 3 mths
	HC	25 (18/7)	25.7 ± 6.27	164.2 ± 7.04	55.2 ± 8.99	–	–	–
Yahia et al.^15^	cLBP	30 (24/6)	41.1 ± 10.9	160.0 ± 6.0	68.0 ± 14.1	3.8 ± 1.1 (VAS)	14.5 ± 4.3 (ODI)	29.9 ± 28.2 mths
	HC	30 (24/6)	39.1 ± 8.9	160.0 ± 7.0	70.5 ± 10.6	–	–	–
Zhang et al.^41^	cLBP	68 (47/21)	30.1 ± 9.0	164.5 ± 7.5	58.7 ± 9.5	5.8 ± 1.3 (VAS)	14.4 ± 7.0 (%: ODI)	38.5 ± 1.3 mths
	HC	40 (26/14)	28.7 ± 7.6	165.9 ± 8.7	57.3 ± 9.8	–	–	–
Total	cLBP	345 (153/98)	35.4 ± 8.2	169.3 ± 5.9	69.9 ± 8.5	4.8 ± 1.1	–	3–139 mths
	HC	318 (115/63)	32.7 ± 6.4	167.8 ± 4.4	65.7 ± 6.7	–	–	–
	Total	663 (268/161)	34.1 ± 7.3	168.6 ± 5.2	67.8 ± 7.8	4.8 ± 1.1	–	3–139 mths

All values in the table except the sample size are represented by means ± standard deviations. cLBP, the chronic low back pain; HC, the healthy control group; F/M, females and males respectively; yrs, years; mths, months; VAS, the visual analogue scale of low back pain; NRS, the number rating scale of low back pain; ODI, Oswestry disability index questionnaire; RM, Roland-Morris disability questionnaire.

### Study characteristics

Table 2 shows information extracted from the 16 included studies, including which questionnaires/scales were implemented, the methods used to collect CoP data, the types of quiet standing tasks, and whether significant differences were found between cLBP and HC groups. Across all studies, pain intensity was most often assessed using a VAS and disability was most often assessed using the Oswestry disability index (ODI). Three studies did not report pain intensity^22,23,40^. Eight studies reported disability for pain using ODI^15,21–23,39,41–43^, and four studies used the Roland Morris disability questionnaire (RM)^13,14,20,24^. Four studies did not assess disability^6,38,40,44^. Anxiety, cognitive function, and health condition questionnaires are also included in Table 2 if included in the corresponding study. For the measurement of CoP variables, the sampling rate ranged from 40 to 1500 Hz. Eight studies used 100 Hz^13,14,20–22,24,38,42^, and two studies did not report the sampling rates^23,44^. A Butterworth filter was used for all studies with cut-off frequencies ranging from 2.5 to 35 Hz and eight studies not reporting cut-off frequencies used^14,15,23,38–41,44^. The duration of each trial between studies varied from 10 to 120 s. Six articles used 30 s^6,14,20,38,39,41^, and one study did not report trial duration^44^. Most studies (5 studies) used three trials of quiet standing^13,20,22,23,39^, and three articles did not report the number of trials^6,15,42^. A total of seven studies gave subjects instructions for quiet standing tasks^15,21–23,41–43^. Among them, five studies instructed subjects to stand as still as possible^15,21,23,41,43^. Two studies instructed subjects to relax^22^ and to be comfortable^42^ during quiet standing. The remaining nine studies did not report their instruction for subjects^6,13,14,20,24,38–40,44^. For task conditions, CoP movement was measured on a firm surface with eyes open in 16 studies^6,13–15,20–24,38–44^ and on a firm surface with eyes closed in 13 studies^6,13–15,20,22,23,38–42,44^. On a foam surface, four studies assessed CoP movement with eyes open^13–15,44^ and four studies measured CoP with eyes closed^13–15,44^. The last column of Table 2 shows the significant differences between cLBP and HC groups. Twelve studies found CoP movement was greater or faster in cLBP group as compared to HC group^6,13–15,20–22,38–41,44^, while one study found that CoP movement was smaller or slower in cLBP group than HC group^21^. Four studies did not find significant between group differences^23,24,42,43^.

**Table 2:** Characteristics of studies in the meta-analysis.

Authors	Questionnaires	Measurement	Task condition	Results (Significant differences)
Abbasi et al.^42^	ODI NRS	Force plate Sampling rate (100 Hz) Low cut-off frequency (6 Hz) Task duration (20 s) # Trials (-) Arms are on their side body Stand comfortably with normal posture	Firm EO	–
			Firm EC	–
Caffaro et al.^13^	RM VAS SF36-physical aspect SF36-emotion	Balance master Sampling rate (100 Hz) Low cut-off frequency (8 Hz) Task duration (10 s) # Trials (3) Arms are on their side body No instruction for tasks	Firm EO	–
			Firm EC	–
			Foam EO	–
			Foam EC	Total oscillation (AP-ML), RMS (AP), Area (AP-ML), and Vel (AP-ML): cLBP > HC
Cana-Pino et al.^14^	RM NRS TSK	Pressure platform Sampling rate (100 Hz) Low cut-off frequency (-) Task duration (30 s) # Trials (1) Arms are on their side body No instruction for tasks	Firm EO	Displacement (AP): cLBP > HC
			Firm EC	Displacement (AP and ML): cLBP > HC
			Foam EO	Displacement (AP): cLBP > HC
			Foam EC	Displacement (AP): cLBP > HC
da Silva et al.^20^	RM VAS FABQp FABQw	Force plate Sampling rate (100 Hz) Low cut-off frequency (35 Hz) Task duration (30 s) # Trials (3) Arms are on their side body No instruction for tasks	Firm EO	Area (AP-ML): cLBP > HC
			Firm EC	Area (AP-ML): cLBP > HC
della Volpe et al.^23^	ODI	Neurocom Sampling rate (-) Low cut-off frequency (-) Task duration (20 s) # Trials (3) Arms are on their side body Stand as still as possible	Firm EO	–
			Firm EC	–
Hamaoui & Bouisset^40^		Force plate Sampling rate (50 Hz) Low cut-off frequency (-) Task duration (20 s) # Trials (5) Arms are on their side body No instruction for tasks	Firm EO	–
			Firm EC	Sway amp (AP): cLBP > HC
Lafond et al.^21^	ODI VAS FABQ	Force plate Sampling rate (100 Hz) Low cut-off frequency (10 Hz) Task duration (60 s) # Trials (1) Arms are on their side body Stand as still as possible	Firm EO	Vel (AP), RMS (AP), and Area (AP and ML): cLBP > HC Vel (ML) and RMS (ML): cLBP < HC
Mann et al.^38^	VAS	Force plate Sampling rate (100 Hz) Low cut-off frequency (-) Task duration (30 s) # Trials (> 1) Arms are on their side body No instruction for tasks	Firm EO	Displacement (AP and ML): cLBP > HC
			Firm EC	Displacement (AP and ML): cLBP > HC Vel (AP-ML): cLBP > HC
Nogueira et al.^6^	VAS	Force plate Sampling rate (1000 Hz) Low cut-off frequency (5 Hz) Task duration (30 s) # Trials (-) Arms are on their side body No instruction for tasks	Firm EO	Sway displacement (AP-ML), Dispersion (ML), Sway amp (ML), Vel (all), and Area (AP-ML): cLBP > HC
			Firm EC	Sway displacement (AP-ML), Dispersion (ML), Sway amp (AP and ML), Vel (all), and Area (AP-ML): cLBP > HC
Popa et al.^22^	ODI	Strain gauge platform Sampling rate (100 Hz) Low cut-off frequency (2.5 Hz) Task duration (20 s) # Trials (3) Arms are on their side body Relaxed standing posture	Firm EO	Displacement (AP-ML): cLBP > HC
			Firm EC	Displacement (AP-ML): cLBP > HC
			Firm EC	–
Ringheim et al.^43^	ODI NRS Tampa	Force plate (AMTI) Sampling rate (1500 Hz) Low cut-off frequency (20 Hz) Task duration (60 s) # Trials (> 1) Arms are on their side body Stand as still as possible	Firm EO	–
Shigaki et al.^24^	RM VAS FABQ	Force plate Sampling rate (100 Hz) Low cut-off frequency (35 Hz) Task duration (120 s) # Trials (2) Arms are on their side body No instruction for tasks	Firm EO	–
Sundaram et al.^44^	VAS	Bertec force plate Sampling rate (-) Low cut-off frequency (-) Task duration (-) # Trials (2) Arms are crossed on their chest No instruction for tasks	Firm EO	Displacement (AP-ML): cLBP > HC
			Firm EC	
			Foam EO	
			Foam EC	
Wang et al.^39^	ODI VAS	Balancing instrument Sampling rate (50 Hz) Low cut-off frequency (-) Task duration (30 s) # Trials (3) Arms are on their side body No instruction for tasks	Firm EO	Displacement (AP-ML): cLBP > HC Area (AP-ML): cLBP > HC
			Firm EC	
Yahia et al.^15^	ODI VAS	SATEL force plate Sampling rate (40 Hz) Low cut-off frequency (-) Task duration (51.2 s) # Trials (-) Arms are on their side body Stand as still as possible	Firm EO	–
			Firm EC	–
			Foam EO	Displacement (AP-ML) and Area (AP-ML): cLBP > HC
			Foam EC	Displacement (ML and AP-ML) and Area (AP-ML): cLBP > HC
Zhang et al.^41^	ODI VAS PCS PCSh PCSm PCSr SFMPQ SFMPQa SFMPQs	PROKIN system Sampling rate (50 Hz) Low cut-off frequency (-) Task duration (30 s) # Trials (2) No instruction for the arm position Stand as still as possible	Firm EO	–
			Firm EC	Area (AP-ML): cLBP > HC

cLBP, chronic low back pain; ODI, Oswestry disability index questionnaire; TSK, Tampa scale of kinesiophobia; HC, healthy control group; VAS, visual analog scale of low back pain; SF36, short-form 36 health survey questionnaire; NRS, the number rating scale of low back pain; FABQ, fear avoidance beliefs questionnaire; FABQp, fear avoidance beliefs questionnaire related to physical activity; FABQw, fear avoidance beliefs questionnaire related to work; RM, Rolland-Morris disability questionnaire; PCS, pain catastrophizing scale; PCSh, helplessness subscale of pain catastrophizing scale; PCSm magnification subscale of pain catastrophizing scale; PCSr, rumination subscale of pain catastrophizing scale; SFMPQ, short-from McGill pain questionnaire; SFMPQa, affective subscale in short-form McGill pain questionnaire; SFMPOs, sensory subscale in short-form McGill pain questionnaire; Firm EO, firm (stable) surface with eyes open; Firm EC, firm (stable) surface with eyes closed; Foam EO, foam with eyes open; Foam EC, foam with eyes closed; Vel, velocity; AP, anteroposterior direction; ML, mediolateral direction; AP-ML, sagittal (AP) and horizontal (ML) plane.

### Data handling

From 16 studies, we extracted 111 combinations of means, standard deviation, standard errors, sample sizes, *p*-values, and/or t-values. A total of 58 effect sizes were calculated from 11 studies using a combination of means, standard deviations, and sample sizes^14,15,20,22,24,38–42,44^. A total of 20 effect sizes were calculated from two studies by converting standard errors to standard deviations. A total of five effect sizes were calculated from one study that reported medians and interquartile ranges^43^. In this case, we converted those values to means and standard deviations based on the Quantile estimation method^27^. Four studies did not report means, standard deviations, or both^6,21,38,44^. In this case, we extracted sample size and other statistical values—independent t-values^21^ and *p*-values^6,38,44^—and calculated 28 effect sizes using combinations of these values. Among 111, 81 effect sizes calculated from 15 studies^6,13–15,20–24,38–41,43,44^ were body sway outcomes, and 30 effect sizes calculated from nine studies^6,13,20,21,23,24,38,42,43^ were body sway velocity outcomes. Additional data extracted from each study is shown in Supplementary material 2.

### Pooled effect sizes of CoP movement for body sway and body sway velocity

**Figure 2:**
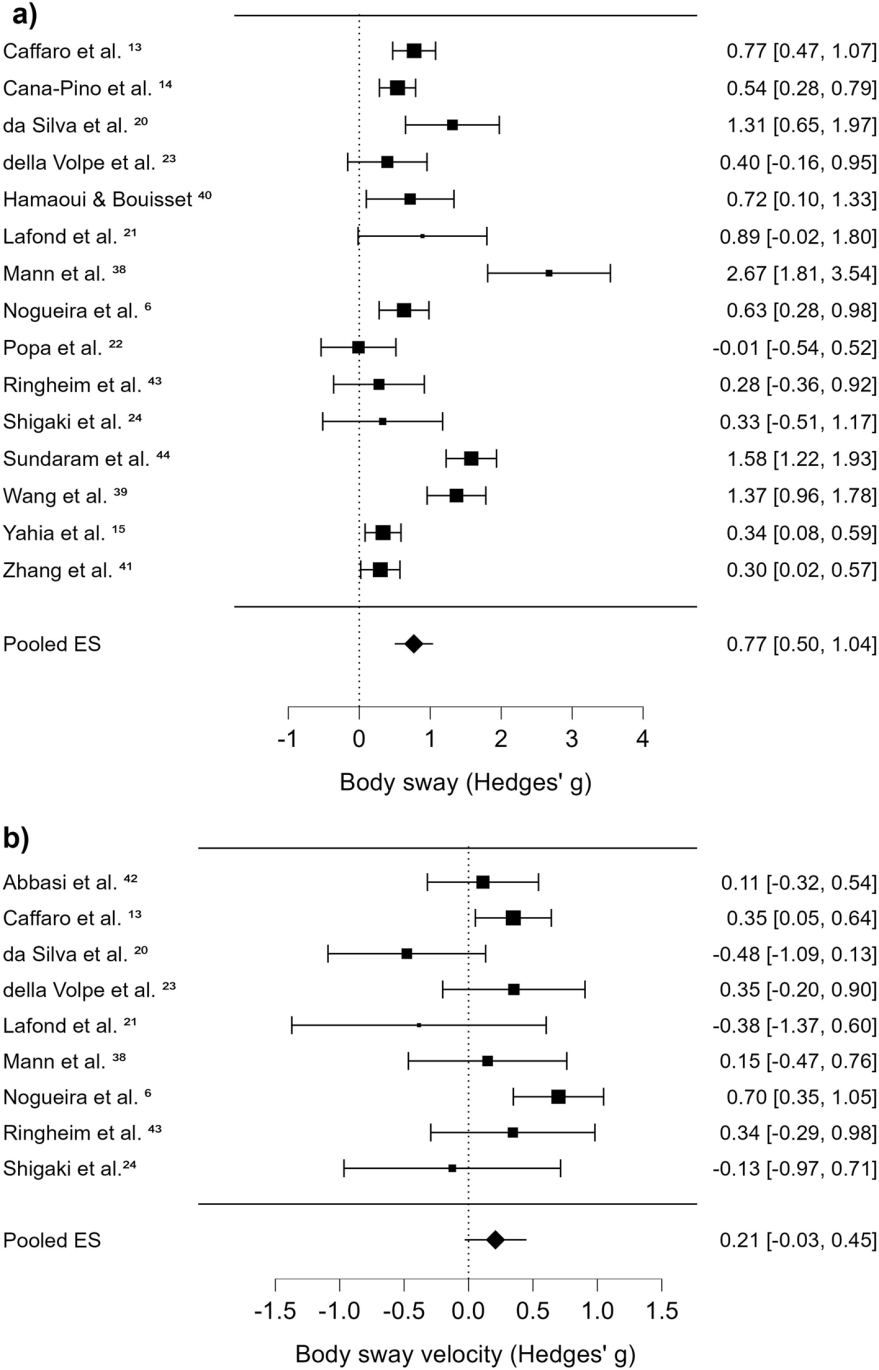
*Forest plots for body sway and body sway velocity. Note*. This forest plot represents meta-analysis results for body sway (**a**) and body sway velocity (**b**). The leftmost column lists the name of studies included in the meta-analysis, and the rightmost column lists effect sizes and 95% confidence intervals for the studies. The effect sizes were calculated into Hedges’ g which is interpreted based on 3 stages: (1) values from 0.2 to 0.49 represent a small effect size; (2) values from 0.5 to 0.79 represent a medium effect size; (3) values greater than or equal to 0.8 represent a large effect size. The black squares represent the locations of the effect sizes for each study, and the whiskers represent the confidence intervals for each study. If a confidence interval is not overlapped with a zero value which is indicated as a dot line, there was a significant effect on body sway/body sway velocity between chronic low back pain (cLBP) and healthy control (HC) groups. In instances where a significant effect size is positive, individuals with cLBP in the study showed bigger magnitude of body sway or body sway velocity compared to HC group. The black diamonds represent pooled effect sizes across studies. For Fig. 2a, the pooled effect size was 0.77, showing a significant, positive, and large effect on body sway. It suggests that individuals with cLBP have greater magnitude of body sway compared to HCs. In contrast, for Fig. 2b, no significant effect on body sway velocity was found.

Figure 2a shows body sway effect sizes between cLBP and HC groups. A total of 15 effect sizes were calculated from 15 studies. Within each study, effect sizes were averaged across all task conditions. Black squares represent effect sizes for each study and the whiskers represent the confidence interval. These values are also shown on the right of the Fig. 2a for each study. For example, the Caffaro et al.^13^ study had an effect size of 0.77, with a confidence interval of 0.47 – 1.07. The pooled effect size is shown at the bottom of Fig. 2a, and was large, positive, and significant, revealing a greater amplitude of CoP body sway in the cLBP group as compared to the HC group (*p* < 0.001, *g* = 0.77 [0.50, 1.04]). The I^2^ value was 83.60% and PI was −0.29 to 1.82 (Table S4). Figure 2b shows body sway velocity effect sizes. The pooled effect size of 0.21 (−0.03, 0.45), was not significant, and heterogeneity was 46.95% (I^2^) and PI was − 0.43 to 0.85 (Table S4).

## Subgroup analyses for body sway

### Quiet standing on a firm surface with eyes open

**Figure 3:**
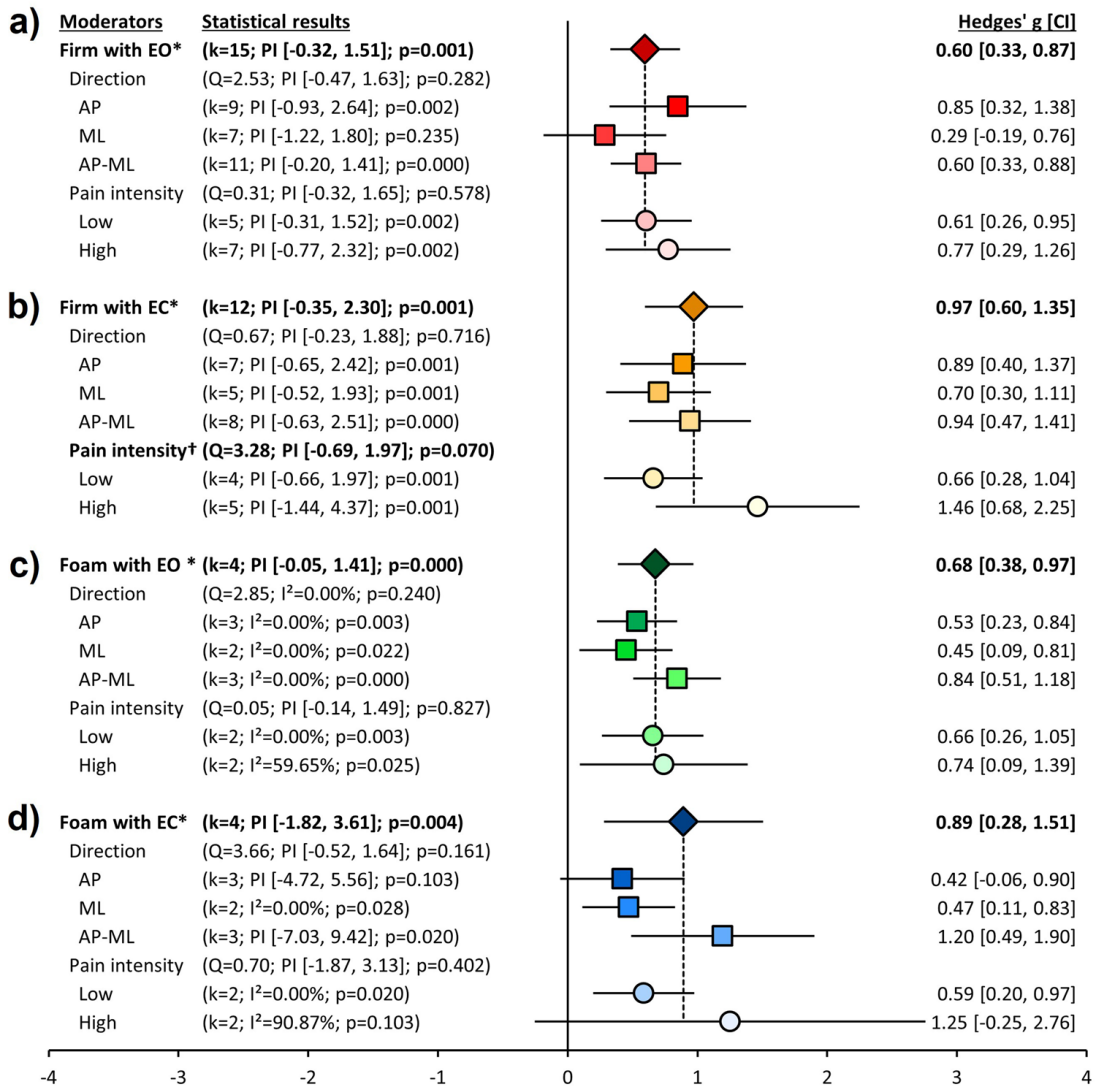
*Subgroup analyses for body sway. Note*. Those forest plots indicate the results of subgroup analyses for body sway according to quiet standing conditions. The leftmost column represents moderate variables: conditions, directions (i.e., anterior–posterior [AP], medial–lateral [ML], and both [AP-ML] directions) to measure center of pressure (CoP), and pain intensity (Low and High pain). The second column represents the statistical results: k, the number of studies; PI, prediction interval; I^2^, heterogeneity; *p*, *p*-values; Q, heterogeneity for moderate variables. The third column represents forest plots for each subgroup analysis: diamonds stand for pooled effect sizes for quiet standing conditions (the firm surface with eyes open [EO; dark red] and eyes closed [EC; dark yellow] and the foam surface with EO [dark green] and EC [dark blue]); the squares stand for the pooled effect sizes for results of CoP directions (AP, ML, and AP-ML); the circles stand for the pooled effect sizes for pain intensity (Low and High). All whiskers represent confidence intervals for each analysis. The rightmost column lists effect sizes and confidence intervals, which were calculated into Hedges’ g. Figure 3a displays results for the firm surface with EO condition (red colors). Figure 3b represents results on quiet standing on the firm surface with EC (yellow and orange colors). Figure 3c represents results on quiet standing on the foam surface with EO (green colors). Figure 3d represents results on quiet standing on the foam surface with EC (blue colors). ^*^p < .05; ^†^*p*_*Q-test*_ < .10.

Figure 3a shows effect sizes for body sway on a firm surface with eyes open (EO). All the effect sizes were positive reflecting greater body sway in the cLBP group. The medium and significant pooled effect size (*p* < 0.001, *g* = 0.60 [0.33, 0.87]) is represented by the red diamond. The I^2^ value was 60.62%, and the PI was from − 0.32 to 1.51 (Table S4). No effect of movement direction was found (Q = 2.53, *p* = 0.282, I^2^ = 60.62%, PI [−0.47, 1.63]). The red squares in Fig. 3a represent effect sizes for AP, ML, and AP-ML directions. A large and significant effect was found in the AP direction (*p* = 0.002, *g* = 0.85 [0.32, 1.38], I^2^ = 81.50%, PI [−0.93, 2.64]) and a medium and significant effect was found in the AP-ML direction (*p* < 0.001, *g* = 0.60 [0.33, 0.88], I^2^ = 52.93%, PI [−0.20, 1.41]). No significant moderator effect was found for pain intensity (Q = 0.31, *p* = 0.578, I^2^ = 64.46%, PI [−0.32, 1.65]). The pink circles in Fig. 3a represent effect sizes for the low and high pain levels. Both pain levels showed medium and significant effects (Low: *p* = 0.002, *g* = 0.61 [0.26, 0.95], I^2^ = 32.92%, PI [−0.31, 1.52]; High: *p* = 0.002, *g* = 0.77 [0.29, 1.26], I^2^ = 75.96%, PI [−0.77, 2.32]).

### Quiet standing on a firm surface with eyes closed

Figure 3b indicates effect sizes for body sway on a firm surface with eyes closed (EC). All the effect sizes were again positive revealing with greater sway in the cLBP group. The large and significant pooled effect size (*p* = 0.001, *g* = 0.97 [0.60, 1.35]) is represented by the orange diamond. The I^2^ value was 75.66%, and the PI was −0.35 to 2.30 (Table S4). No effect of movement direction was found (Q = 0.67; *p* = 0.716, I^2^ = 71.49%, PI [−0.23, 1.88]). The yellow squares represent sway in the AP, ML, and AP-ML directions. Large and significant effects were found for the AP and AP-ML directions (AP: *p* = 0.001, *g* = 0.89 [0.40, 1.37], I^2^ = 73.40%, PI [−0.65, 2.42]; AP-ML: *p* = 0.001, *g* = 0.94 [0.47, 1.41], I^2^ = 79.88%, PI [−0.63, 2.51]). A medium and significant effects was found for the ML direction, (*p* = 0.001, *g* = 0.70 [0.30, 1.11], I^2^ = 51.92%, PI [−0.52, 1.93]). A significant moderator effect was found for pain intensity (Q = 3.28; *p* = 0.070, I^2^ = 80.43%, PI [−0.69, 1.97]), with the large effect (*p* = 0.001, *g* = 1.46 [0.68, 2.25], I^2^ = 87.44%, PI [−1.44, 4.37]) driven by greater body sway in individuals with high pain compared to controls (i.e., the lighter yellow circle in Fig. 3b). In the low pain level represented by the darker yellow circle in Fig. 3b, a medium and significant effect was found (*p* = 0.001, *g* = 0.66 [0.28, 1.04], I^2^ = 38.16%, PI [−0.66, 1.97]).

### Quiet standing on a foam surface with eyes open

Figure 3c shows effect sizes for body sway on a foam surface with EO. All effect sizes were positive in favor of cLBP group. A medium and significant pooled effect size was found (*p* < 0.001, *g* = 0.68 [0.38, 0.97]) which is represented by the green diamond. I^2^ value was 7.55%, and the PI was −0.05 to 1.41 (Table S4). No effect of movement direction (Q = 2.85; *p* = 0.240, I^2^ = 0%, PI = NaN) was found. The green squares in Fig. 3c indicate AP, ML, and AP-ML, showing significant effects: AP had the medium effect size (*p* = 0.003, *g* = 0.53 [0.23, 0.84], I^2^ = 0%, PI = NaN); ML had the small effect size (*p* = 0.022, *g* = 0.45 [0.09, 0.81], I^2^ = 0%, PI = NaN); AP-ML represented by the lighter square had a large effect size (*p* < 0.001, *g* = 0.84 [0.51, 1.18], I^2^ = 0%, PI = NaN). A moderator effect of pain intensity (Q = 0.05; *p* = 0.827, I^2^ = 7.55%, PI [−0.14, 1.49]) was not found. The green circles in Fig. 3c represent medium and significant effects for both pain levels (Low: *p* = 0.003, *g* = 0.66 [0.26, 1.05], I^2^ = 0%, PI = NaN; High: *p* = 0.025, *g* = 0.74 [0.09, 1.39], I^2^ = 59.65%, PI = NaN).

### Quiet standing on a foam surface with eyes closed

Figure 3d represents effect sizes for body sway on a foam surface with EC. All effect sizes were positive in favor of cLBP group. The large and significant pooled effect size (*p* = 0.004, *g* = 0.89 [0.28, 1.51]) is represented by the blue diamond. The I^2^ value was 77.42%, and the PI was −1.82 to 3.61 (Table S4). No moderator effect of movement direction was found (Q = 3.66; *p* = 0.161, I^2^ = 69.04%, PI [−0.52, 1.64]). The blue squares represent AP, ML, and AP-ML. The ML plane had a small and significant effect (*p* = 0.028, *g* = 0.47 [0.11, 0.83], I^2^ = 0%, PI = NaN), and the AP-ML plane had a large and significant effect (*p* = 0.020, *g* = 1.20 [0.49, 1.90], I^2^ = 74.28%, PI [− 7.03, 9.42]). We found no evidence of a moderator effect of pain intensity (Q = 0.70; *p* = 0.402, I^2^ = 77.42%, PI [− 1.87, 3.13]). The blue circles in Fig. 3d indicate low and high pain levels. A significant medium effect of the low pain level was found (*p* = 0.020, *g* = 0.59 [0.20, 0.97], I^2^ = 0%, PI = NaN) represented by the darker blue circle.

### Subgroup analyses for body sway velocity

**Figure 4:**
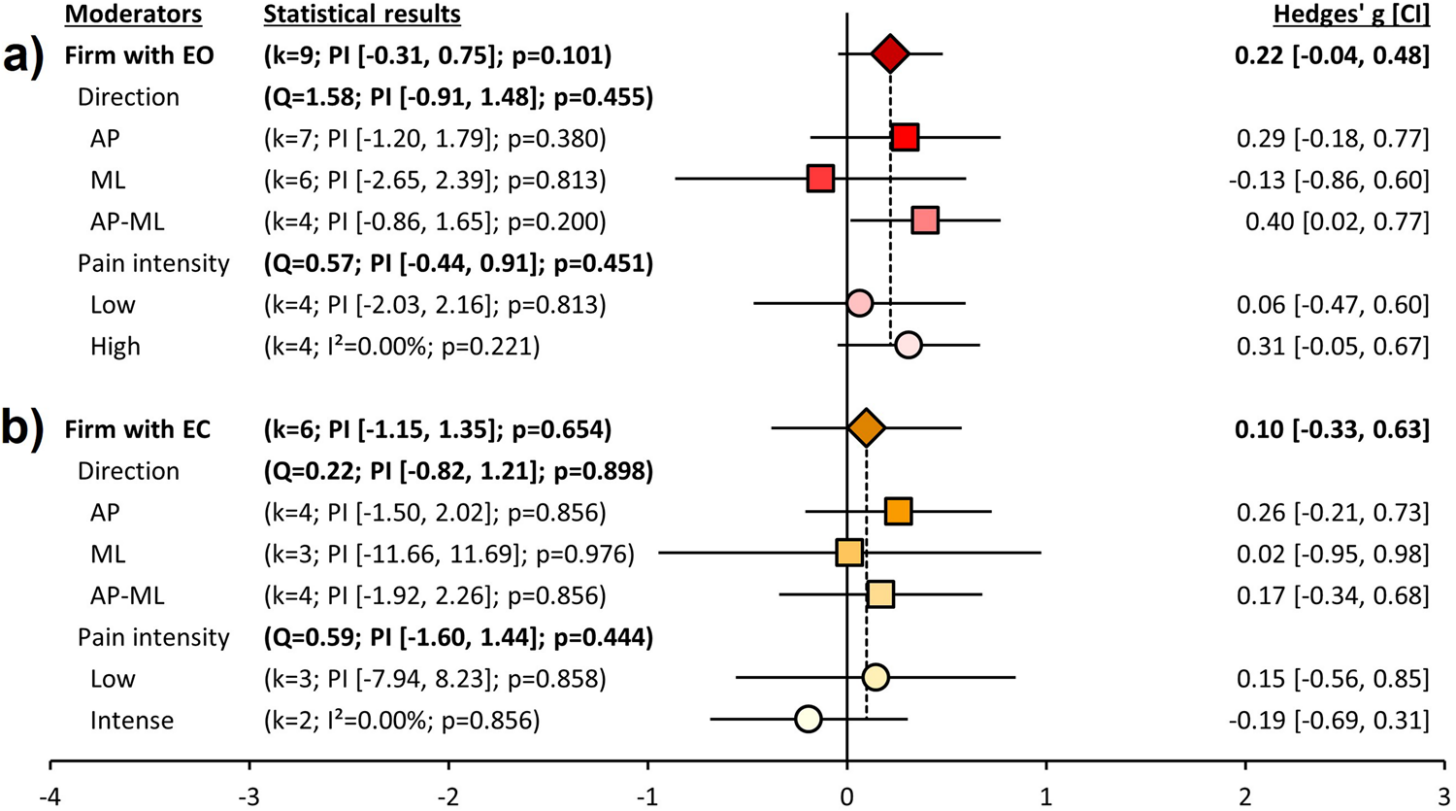
*Subgroup analyses for body sway velocity. Note*. Those forest plots indicate the results of subgroup analyses for body sway velocity according to quiet standing conditions. The leftmost column represents moderate variables: conditions, directions (i.e., anterior–posterior [AP], medial–lateral [ML], and both [AP-ML] directions) to measure center of pressure (CoP), and pain intensity (self-reported Low and High pain). The second column represents the statistical results: k, the number of studies included in each subgroup analysis; PI, prediction interval; I^2^, heterogeneity for each subgroup analysis; *p*, *p*-values for each subgroup analysis; Q, heterogeneity for moderate variables. The third column represents forest plots for each subgroup analysis: diamonds stand for pooled effect sizes for quiet standing conditions (the firm surface with eyes open [EO] and eyes closed [EC]); the squares stand for the pooled effect sizes for results of CoP directions (AP, ML, and AP-ML); the circles stand for the pooled effect sizes for pain intensity (Low and High). All whiskers stand for confidence intervals for each analysis. The rightmost column represents effect sizes and confidence intervals, which were calculated into Hedges’ g. Figure 4a indicates results on the condition on firm surface with EO (red colors). Figure 4b represents results on quiet standing on the firm surface with EC (yellow and orange colors). ^*^p < .05; ^†^*p*_*Q-test*_ < .10.

Figure 4 indicates effect sizes for body sway velocity. No significant pooled effect size or moderator effect was found as shown in Fig. 4a and 4b.

### Publication bias

Publication biases were assessed using Egger’s regression and Trim and Fill analysis. Table S5 in the supplementary material 1 represents the statistical results of Egger’s regression test. The last column shows *p*-values for all meta- and subgroup analyses. Significant biases were found in the three conditions of body sway: the firm with EC (*p* = 0.033) and foam with EO (*p* = 0.006) and EC (*p* = 0.033). Those analyses were reassessed using the Trim and Fill analysis.

Fig. S2 in the supplementary material 1 represents the results of the Trim and Fill analyses. The circles in the funnel plots represent standard errors by Hedges’ g of each study. The white filled diamond under the x-axis represents the overall 95% confidence interval for each meta-analysis. For instance, Fig. S2a shows the funnel plot for the body sway meta-analysis when collapsing across all conditions within each study. Each study is represented by an open circle. The absence of a filled black circle means that no imputation had to be conducted for this meta-analysis. Open circles were evident for the firm surface with EO (Fig. S2b) and firm surface with EC (Fig. S2c). Fig. S2d and e show the funnel plots for body sway on a Foam surface with EO and EC. Black circles are included in each plot. Each black circle represents one imputed study, which makes the distribution of the values symmetric. The black diamond on the x-axis represents the mean confidence interval when including the imputed data. When the white and black diamonds overlap, we infer that publication bias is not affected. This was evident in each case. Figs. S2f–h show funnel plots for body sway velocity meta-analyses. Although black dots are shown in each case, overlap between the white and black diamond suggests no evidence of publication bias in either case.

### Assessment of the quality in studies

The results of QAT for every study are presented in Table S3. Five studies were evaluated as being good quality^13,14,39,41,43^, and six studies were of fair quality^15,20–24^. Five studies were rated as poor quality^6,38,40,42,44^.

### GRADE assessment

GRADE was used to assess the evidence quality and the results are shown in Table 3. The first column in Table 3 lists the different outcome measures and conditions, and the second column represents the number of studies included in the corresponding meta-analysis as well as the total sample size and CoP variables included. The third to seventh columns indicate the ratings for subcategories of GRADE. The eight column (“other”) shows decisions on publication bias and other considerations that can upgrade the quality of evidence, which is followed by estimate of outcome values. The final column represents the final decision for rating evidence certainty. As a result, the evidence of CoP outcomes on Firm with EC in body sway, CoP outcome in body sway velocity, and CoP outcome on Firm with EO in body sway velocity were rated as low certainty. The rest of evidence was rated as very low certainty.

**Table 3:** GRADE assessment for the certainty of evidence.

Quality assessment							Estimate of outcomes (g, CI, PI)	Certainty
Outcomes	Study design/ measurement instrument	Risk of Bias	Inconsistency	Indirectness	Imprecision	Other		
Body sway	15 studies, 623 subjects CoP of area, dispersion, displacement, sway amplitude	No serious risk of bias	Serious inconsistency ^a^	No serious indirectness	No serious imprecision	None	g: 0.77 CI: 0.50–1.04 PI: -0.29, 1.82	⊕ ◯◯◯ Very low
Firm EO	15 studies, 623 subjects CoP of area, dispersion, displacement, sway amplitude	No serious risk of bias	Serious inconsistency ^a^	No serious indirectness	No serious imprecision	None	g: 0.60 CI: 0.33, 0.87 PI: -0.32, 0.75	⊕ ◯◯◯ Very low
Firm EC	12 studies, 542 subjects CoP of area, dispersion, displacement, sway amplitude	No serious risk of bias	Serious inconsistency ^a^	No serious indirectness	No serious imprecision	High effect ^b^	g: 0.97 CI: 0.60, 1.35 PI: -0.35, 2.30	⊕ ⊕ ◯◯ Low
Foam EO	4 studies, 204 subjects CoP of area, displacement, sway amplitude	No serious risk of bias	No serious inconsistency	No serious indirectness	Serious imprecision ^c^	None	g: 0.68 CI: 0.38, 0.97 PI: -0.05, 1.41	⊕ ◯◯◯ Very low
Foam EC	4 studies, 204 subjects CoP of area, displacement, sway amplitude	No serious risk of bias	Serious inconsistency ^a^	No serious indirectness	Serious imprecision ^c^	High effect ^b^	g: 0.89 CI: 0.28, 1.51 PI: -1.82, 3.61	⊕ ◯◯◯ Very low
Body sway velocity	9 studies, 294 subjects CoP of velocity	No serious risk of bias	No serious inconsistency	No serious indirectness	No serious imprecision	None	g: 0.21 CI: -0.03, 0.45 PI: -0.43, 0.85	⊕ ⊕ ◯◯ Low
Firm EO	9 studies, 294 subjects CoP of velocity	No serious risk of bias	No serious inconsistency	No serious indirectness	No serious imprecision	None	g: 0.22 CI: -0.04, 0.48 PI: -0.31, 0.75	⊕ ⊕ ◯◯ Low
Firm EC	6 studies, 213 subjects CoP of velocity	No serious risk of bias	Serious inconsistency ^a^	No serious indirectness	Serious imprecision ^c^	None	g: 0.10 CI: -0.33, 0.63 PI: -1.15, 1.35	⊕ ◯◯◯ Very low

The Grading of Recommendations Assessment, Development and Evaluation (GRADE) assesses certainty of evidence body based on four criteria: High, Moderate, Low, and Very low certainty. The High certainty represents very confidence that the true effect lies close to that of the estimate of the effect; the Moderate certainty represents moderately confidence that the true effect is likely to be close to the estimated effect; the Low certainty represents limited confidence in the estimate of the effect; the Very low certainty represents very little confidence in an estimated effect.

*Firm EO* The firm surface with eyes open; *Firm EC* The firm surface with eyes closed; *Foam EO* The foam surface with eye open; *Foam EC* The foam surface with eyes closed; *CoP* Center of pressure; g, Hedges’ g; *CI* confidence intervals; *PI* prediction intervals.

^a^The I^2^ was higher than 50% that means a high level of heterogeneity.

^b^The result of this meta-analysis showed a high and pooled effect size. In this case, we upgraded the level of certainty of evidence^36^.

^c^The sample size for the meta-analysis was lower than an optimal sample size (n = 244) calculated based on 5% of margin of error, 95% CI, and the total sample size (n = 663).

## Discussion

Compared with controls, individuals with cLBP showed increased postural sway during quiet standing. Subgroup analyses revealed larger effect sizes when vision was removed and when self-reported pain intensity was higher but only when subjects completed the task on a firm surface. In contrast, we did not find strong support for an effect of cLBP on postural sway velocity.

When averaging data across all experimental conditions within each study, we found strong evidence that cLBP is associated with greater postural sway during quiet standing. Our findings suggest that cLBP is associated with altered postural control^7,45^. Several explanations may account for this change in control including reduced proprioceptive acuity^46^, restrictive trunk movement^5^, and protective trunk muscle strategies^47,48^. Reduced proprioception means that larger movements must be made for a change in internal estimate of CoP position to be updated^11^. Once perceived, a correction in the opposite direction may also then be disproportionality large^22^. Restrictive trunk movement and co-contractions across trunk muscles may also contribute to changes in posture by shifting control from the trunk to the ankles. LBP has been associated with co-activation of left and right gluteus medius muscles^49,50^, sustained activation of back and abdominal muscles during quiet standing^43^, leading to a restriction in trunk movement reflective of a protective strategy^12,47^. In addition to changes in trunk muscle activation, a shift in motor control away from the trunk has also been evidenced, with the ankle being most commonly observed^5,19,51,52^. This shift in control to the ankle can be problematic given that perturbation studies show that trunk control is most effective for maintaining postural equilibrium^53^, with the ankles contributing minimally to posture. Taken together, the greater CoP sway in cLBP evident in our findings may result from altered proprioception and restrictive and protective trunk strategies, which combine to shift postural control to the ankles.

Previous systematic reviews on back pain and postural sway have reported inconsistent results^5,18,54^. In the current meta-analysis twelve out of 16 articles reported greater CoP sway in cLBP compared to healthy controls. The difference in findings between previous reviews and the present meta-analysis may be due to different inclusion criteria for the back pain group. Our meta-analysis included studies that recruited individuals with cLBP, while previous reviews included individuals with both recurrent and cLBP^5,16,18^. Smaller CoP movements have been reported in LBP studies (e.g., Mok et al.^55^ and Salavati et al.^56^), these were excluded from our meta-analysis because they did not meet our inclusion criteria for cLBP. Models of motor adaptation have been proposed for muscular pain^7,57,58^, such that motor strategies are altered based on pain chronicity. For instance, the progression from acute to chronic neck-shoulder pain is associated with a progressive decrease in variability of trunk movement with a corresponding increase trunk stiffness^57^. In the context of LBP, it is plausible, therefore, that acute LBP is associated with an increase in variability of trunk movement and a decrease in trunk stiffness as individuals search for optimal solutions to minimize the pain. This could potentially lead to reduced postural sway. As chronicity develops, a progressive decrease in variability and increase in stiffness would lead to an increase in postural sway. We therefore suggest that distinguishing between acute, recurrent and chronic phases of LBP is critical when assessing the impact of low back pain on postural sway.

Removing vision during quiet standing increased effect sizes by ~ 0.2 irrespective of which surface individuals were standing on. Effect sizes were similar for both eyes open conditions (0.64 and 0.68) and both eyes closed conditions (0.98, 0.89). Changing the surface on which subjects were standing had a minimal impact on effect size. If a deficit in proprioception is a dominant factor in altered postural control in cLBP, then one would have expected effect sizes to be largest in the eyes closed plus foam surface condition. This was not the case in our data, suggesting that the change in surface had a minimal impact. We offer three explanations. First, the number of studies contributing to the foam condition was smaller as compared to the firm condition, and second, altered proprioceptive feedback in cLBP may have reached a ceiling in the eyes open plus firm surface condition (effect size ~ 0.6), such that changing the surface to foam had a minimal additional effect. Other factors, such as deconditioning in cLBP^59^, pain-related fear, and catastrophizing^60^, may also be associated with greater sway, but we did not assess these factors in the current study. In contrast to the surface condition, changing visual input had a larger impact on effect size. Our finding is consistent with other studies in chronic pain that have manipulated vision^61–63^. For instance, individuals with cLBP show greater variability in stride time and minimum foot clearance during gait when vision was removed^61^. Other evidence comes from individuals with chronic neck pain^62,63^. Underestimating the extent of neck rotation using virtual reality led to greater movement prior to the onset of pain^62^. During standing, greater body sway was found in individuals with chronic neck pain compared to controls in eyes open condition, with the between group difference becoming larger when vision was removed^63^.

With eyes closed, we also found evidence that effect sizes were greatest when comparing controls and individuals who self-reported higher as compared to lower pain intensity. This effect was not evident in the eyes open conditions, suggesting that vision may mask or compensate for the effect of pain intensity on postural sway. Our findings are consistent with other studies that have found that individuals who report higher pain intensity (VAS ≧ 5) show greater CoP sway compared to individuals with lower pain intensity (VAS = 1.47)^64^ and controls^14,38,39,41^. Importantly, individuals with lower LBP intensity (VAS < 2) were found to have smaller and slower CoP sway compared to healthy controls^55,56^, which suggests that pain and intensity in addition to pain chronicity should be considered when associating cLBP with postural sway.

We note several limitations of this meta-analysis. First, we restricted our analysis to studies which recruited individuals with cLBP since previous evidence revealed that there is a different impact of the LBP disease stages on a postural control strategy^7,57,58^. Our findings, therefore, can only generalize to individuals with cLBP. Second, we did not consider the impact of specific vs. non-specific cLBP on postural sway. Third, we identified 16 studies that included an eyes-open condition, but the number of studies included in the different analyses and sub-analyses decreased. Fourth, the certainties of the outcomes included in our meta-analysis were rated as low or very low. In part this is because all included studies were observational studies and by default these are initially rated as low certainty. A high magnitude of heterogeneity across CoP outcomes also contributed to the low or very low ratings. High heterogeneity in body sway has been noted in previous systematic reviews^16–18^. We move the field forward by demonstrating that a small portion of the heterogeneity can be accounted for by pain intensity. Nevertheless, we acknowledge that even when accounting for pain intensity and CoP direction, there was still a considerable amount of variance unexplained. Other factors such as different sampling rates, filters, trial durations, number of trial repetitions, CoP equations, and instructions for the quiet standing tasks may help explain this variance, but our sample size was not large enough to address these potential factors. As additional studies are published, future meta-analyses can address these important factors. Nevertheless, we adhered to strict inclusion guidelines and our findings do suggest that vision and pain intensity play important roles in the impact of cLBP on postural sway.

## Conclusion

Impact of this meta-analysis is that cLBP is associated with greater sway, which likely comes from a visual occlusion and a change in control strategy. Interventions that target both of these factors should therefore be explored.

## Supplementary Information

Supplementary Information 1.

Supplementary Information 2.

## Data Availability

All data generated or analyzed during this study are included in this published article (and its Supplementary Information files).
