# Supplementary material for: The effect of chronic low back pain on postural control during quiet standing: A meta-analysis
Source: Sci Rep. 2023 May 16;13:7928. doi: 10.1038/s41598-023-34692-w (PMC10188550; doi:10.1038/s41598-023-34692-w)
Supplement: Supplementary file 1 — Supplementary Information 1. [file 41598_2023_34692_MOESM1_ESM.docx]

**The effect of chronic low back pain on postural control quiet standing: A meta-analysis**

Jinhan Park, Quang Vinh Nguyen, Rachel L. M. Ho, and Stephen A. Coombes.

**Appendix A. Supplemental materials**

Contents

[Table S1. The PRISMA check list 2](#_Toc128470932)

[Key-word strategy to find studies 5](#_Toc128470933)

[Fig. S1. PRISM flow chart for the first search. 6](#_Toc128470934)

[Fig. S2. Funnel plots of Duval and Tweedie’s Trim and Fill 13](#_Toc128470935)

[Table S2. Results of the quality assessment tool for observational cohort and cross-sectional studies. 17](#_Toc128470936)

[Table S3. Results of heterogeneity using the Q-test, Tau^2^, I^2^, and prediction interval. 19](#_Toc128470937)

[Table S4. Results of publication of biases using Egger’s regression test. 21](#_Toc128470938)

## Table S1. The PRISMA check list.

| **Section and Topic** | **Item #** | **Checklist item** | **Location where item is reported** |
| --- | --- | --- | --- |
| **TITLE** | | |  |
| Title | 1 | Identify the report as a systematic review. | Page 1 |
| **ABSTRACT** | | |  |
| Abstract | 2 | See the PRISMA 2020 for Abstracts checklist. | Page 1 |
| **INTRODUCTION** | | |  |
| Rationale | 3 | Describe the rationale for the review in the context of existing knowledge. | Pages 2-3 |
| Objectives | 4 | Provide an explicit statement of the objective(s) or question(s) the review addresses. | Page 3 |
| **METHODS** | | |  |
| Eligibility criteria | 5 | Specify the inclusion and exclusion criteria for the review and how studies were grouped for the syntheses. | Pages 4-5 |
| Information sources | 6 | Specify all databases, registers, websites, organisations, reference lists and other sources searched or consulted to identify studies. Specify the date when each source was last searched or consulted. | Page 4 |
| Search strategy | 7 | Present the full search strategies for all databases, registers and websites, including any filters and limits used. | Page 4 and Page 5 in Supplementary materials 1 |
| Selection process | 8 | Specify the methods used to decide whether a study met the inclusion criteria of the review, including how many reviewers screened each record and each report retrieved, whether they worked independently, and if applicable, details of automation tools used in the process. | Pages 4-5 |
| Data collection process | 9 | Specify the methods used to collect data from reports, including how many reviewers collected data from each report, whether they worked independently, any processes for obtaining or confirming data from study investigators, and if applicable, details of automation tools used in the process. | Pages 4-5 |
| Data items | 10a | List and define all outcomes for which data were sought. Specify whether all results that were compatible with each outcome domain in each study were sought (e.g. for all measures, time points, analyses), and if not, the methods used to decide which results to collect. | Page 5 |
|  | 10b | List and define all other variables for which data were sought (e.g. participant and intervention characteristics, funding sources). Describe any assumptions made about any missing or unclear information. | Pages 5-6 |
| Study risk of bias assessment | 11 | Specify the methods used to assess risk of bias in the included studies, including details of the tool(s) used, how many reviewers assessed each study and whether they worked independently, and if applicable, details of automation tools used in the process. | Pages 6-7 |
| Effect measures | 12 | Specify for each outcome the effect measure(s) (e.g. risk ratio, mean difference) used in the synthesis or presentation of results. | Page 6 |
| Synthesis methods | 13a | Describe the processes used to decide which studies were eligible for each synthesis (e.g. tabulating the study intervention characteristics and comparing against the planned groups for each synthesis (item #5)). | Pages 5, 7 |
|  | 13b | Describe any methods required to prepare the data for presentation or synthesis, such as handling of missing summary statistics, or data conversions. | Page 5 |
|  | 13c | Describe any methods used to tabulate or visually display results of individual studies and syntheses. | Pages 9-15 |
|  | 13d | Describe any methods used to synthesize results and provide a rationale for the choice(s). If meta-analysis was performed, describe the model(s), method(s) to identify the presence and extent of statistical heterogeneity, and software package(s) used. | Pages 7-8 |
|  | 13e | Describe any methods used to explore possible causes of heterogeneity among study results (e.g. subgroup analysis, meta-regression). | Page 6 |
|  | 13f | Describe any sensitivity analyses conducted to assess robustness of the synthesized results. | Pages 7-9 |
| Reporting bias assessment | 14 | Describe any methods used to assess risk of bias due to missing results in a synthesis (arising from reporting biases). | Pages 7-8 |
| Certainty assessment | 15 | Describe any methods used to assess certainty (or confidence) in the body of evidence for an outcome. | Page 8-9 |
| **RESULTS** | | |  |
| Study selection | 16a | Describe the results of the search and selection process, from the number of records identified in the search to the number of studies included in the review, ideally using a flow diagram. | Page 9 |
|  | 16b | Cite studies that might appear to meet the inclusion criteria, but which were excluded, and explain why they were excluded. | Table S2 in the Supplementary material 1 |
| Study characteristics | 17 | Cite each included study and present its characteristics. | Pages 9-11 and Table 2 |
| Risk of bias in studies | 18 | Present assessments of risk of bias for each included study. | Page 16 |
| Results of individual studies | 19 | For all outcomes, present, for each study: (a) summary statistics for each group (where appropriate) and (b) an effect estimate and its precision (e.g. confidence/credible interval), ideally using structured tables or plots. | Figs. 2-4 |
| Results of syntheses | 20a | For each synthesis, briefly summarise the characteristics and risk of bias among contributing studies. | Table 2 and S3 in the Supplementary materials 1 |
|  | 20b | Present results of all statistical syntheses conducted. If meta-analysis was done, present for each the summary estimate and its precision (e.g. confidence/credible interval) and measures of statistical heterogeneity. If comparing groups, describe the direction of the effect. | Pages 12-15 |
|  | 20c | Present results of all investigations of possible causes of heterogeneity among study results. | Pages 13-15 |
|  | 20d | Present results of all sensitivity analyses conducted to assess the robustness of the synthesized results. | Pages 16-17 |
| Reporting biases | 21 | Present assessments of risk of bias due to missing results (arising from reporting biases) for each synthesis assessed. | Page 16 |
| Certainty of evidence | 22 | Present assessments of certainty (or confidence) in the body of evidence for each outcome assessed. | Pages 16-17 and Table 3 |
| **DISCUSSION** | | |  |
| Discussion | 23a | Provide a general interpretation of the results in the context of other evidence. | Pages 17-20 |
|  | 23b | Discuss any limitations of the evidence included in the review. | Pages 20-21 |
|  | 23c | Discuss any limitations of the review processes used. | Pages 20-21 |
|  | 23d | Discuss implications of the results for practice, policy, and future research. | Page 21 |
| **OTHER INFORMATION** | | |  |
| Registration and protocol | 24a | Provide registration information for the review, including register name and registration number, or state that the review was not registered. |  |
|  | 24b | Indicate where the review protocol can be accessed, or state that a protocol was not prepared. |  |
|  | 24c | Describe and explain any amendments to information provided at registration or in the protocol. |  |
| Support | 25 | Describe sources of financial or non-financial support for the review, and the role of the funders or sponsors in the review. |  |
| Competing interests | 26 | Declare any competing interests of review authors. |  |
| Availability of data, code and other materials | 27 | Report which of the following are publicly available and where they can be found: template data collection forms; data extracted from included studies; data used for all analyses; analytic code; any other materials used in the review. |  |

*From:*  Page MJ, McKenzie JE, Bossuyt PM, Boutron I, Hoffmann TC, Mulrow CD, et al. The PRISMA 2020 statement: an updated guideline for reporting systematic reviews. BMJ 2021;372:n71. doi: 10.1136/bmj.n71. <http://www.prisma-statement.org/>

## Key-word strategy to find studies.

**PubMed**

Keywords: (("low back pain"[MeSH Major Topic]) AND ("posture"[MeSH Terms] OR stability[Title/Abstract] OR balance[Title/Abstract] OR "postural sway"[Title/Abstract] OR "postural balance"[MeSH Terms] OR "postural stability"[Title/Abstract] OR COP[MeSH Terms] OR "center of pressure"[Title/Abstract])) NOT ("meta*"[Title/Abstract] OR "systematic review"[Title/Abstract] OR review[Title/Abstract])

**EBSCOhost**

**MEDLINE**, **CINAHL**, **SPORTDiscus**, and **APA** **PsycArticles** were used through EBSCOhost. Keywords: low back pain AND (postural OR stability OR balance OR "postural sway" OR "postural control" OR "postural stability" OR "postural balance" OR COP OR "center of pressure" OR COM OR COG) NOT (meta-analysis OR systematic review OR literature review)

## Fig. S1. PRISM flow chart for the first search.

*
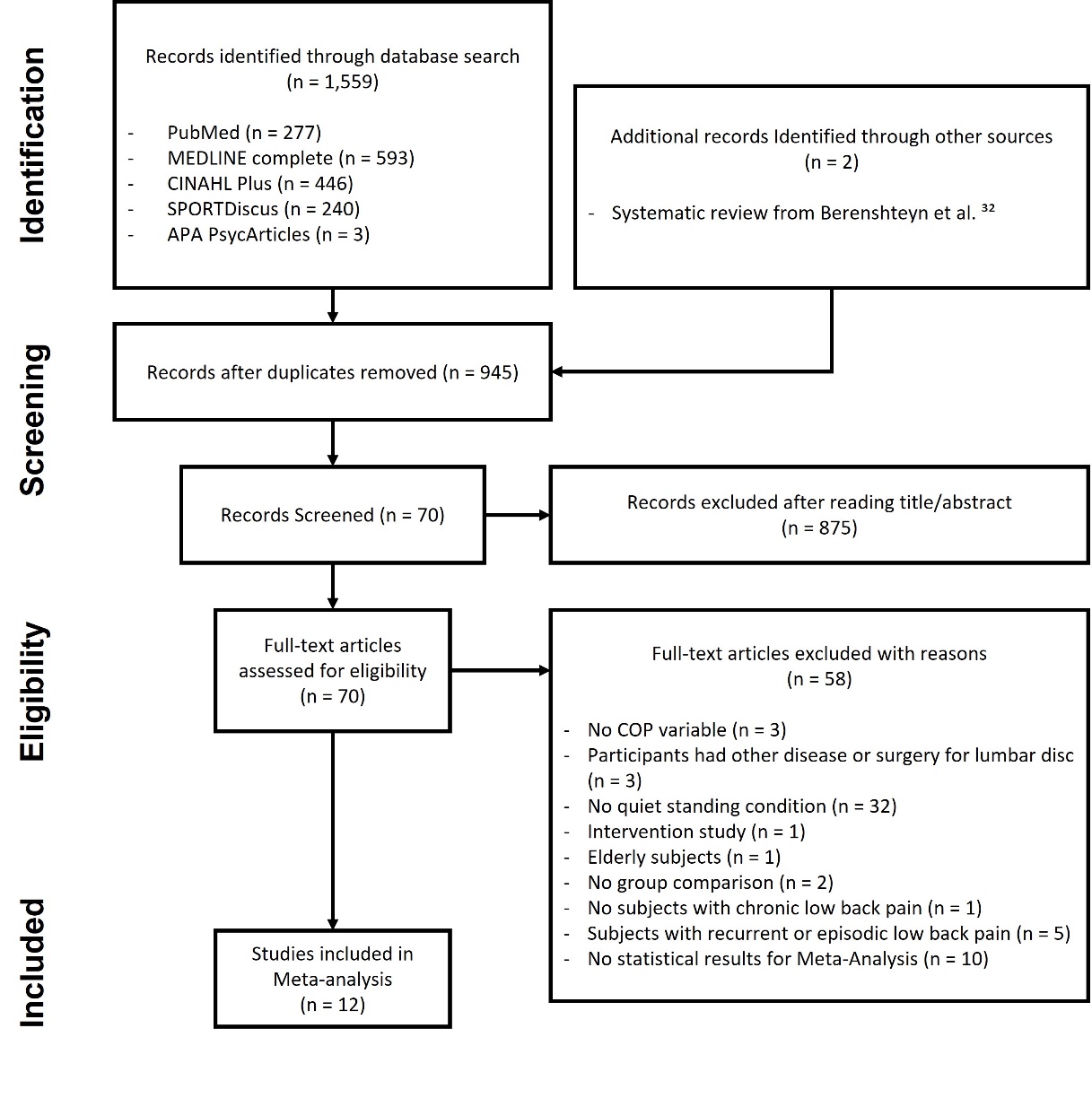
*

## Table S2. Excluded studies.

| Author (year) | Article title | Reason for exclusion |
| --- | --- | --- |
| Abad (2021) | Reliability of postural control during double-leg standing in subjects with nonspecific chronic low back pain: Dual-task paradigm and manipulated visual and somatosensory inputs | No group comparison (no healthy control group) |
| Alexander (1998) | Differences in static balance and weight distribution between normal subjects and subjects with chronic unilateral low back pain | No CoP variable |
| Alsufiany (2020) | Non-specific chronic low back pain and physical activity: A comparison of postural control and hip muscle isometric strength | No CoP variable |
| Ayhan (2016) | Functional contributors to poor movement and balance control in patients with low back pain: A descriptive analysis | No CoP variable |
| Azadinia (2019) | Effects of lumbosacral orthosis on dynamical structure of center of pressure fluctuations in patients with non-specific chronic low back pain: A randomized controlled trial | Subjects with recurrent or episodic low back pain |
| Azadinia (2020) | The amount and temporal structure of center of pressure fluctuations during quiet standing in patients with chronic low back pain | Subjects with recurrent or episodic low back pain |
| Becker (2018) | The relationship between functionality and erector spinae activity in patients with specific low back pain during dynamic and static movements | No CoP variable |
| Bissolotti (2013) | Analysis of differences in postural stability in people with adult scoliosis and non specific low back pain | No a full-text file |
| Bouche (2006) | Comparison of postural control in unilateral stance between healthy controls and lumbar discectomy patients with and without pain | No CoP variable |
| Brumagne (2004) | Proprioceptive weighting changes in persons with low back pain and elderly persons during upright standing | No subjects with chronic low back pain (there is no inclusion criteria for individuals with chronic low back pain |
| Bussey (2020) | Anticipatory postural control differs between low back pain and pelvic girdle pain patients in the absence of visual feedback | No quiet standing condition |
| Celenay (2019) | Immediate effects of kinesio taping on pain and postural stability in patients with chronic low back pain. | No CoP variable |
| Christie (1995) | Postural aberrations in low back pain | Subjects with recurrent or episodic low back pain |
| Ciesielska (2015) | Hip strategy alterations in patients with history of low disc herniation and non-specific low back pain measured by surface electromyography and balance platform | No subjects with chronic low back pain |
| Claeys (2011) | Decreased variability in postural control strategies in young people with non-specific low back pain is associated with altered proprioceptive reweighting | No subjects with chronic low back pain |
| Claeys (2012) | Altered preparatory pelvic control during the sit-to-stance-to-sit movement in people with non-specific low back pain | Subjects with recurrent or episodic low back pain |
| Claeys (2015) | Young individuals with a more ankle-steered proprioceptive control strategy may develop mild non-specific low back pain | No subjects with chronic low back pain |
| Crasto (2020) | Abdominal muscle activity and pelvic motion according to active straight leg raising test results in adults with and without chronic low back pain | No quiet standing condition |
| Daneau (2021) | Impact of load expectations on neuromuscular and postural strategies during a freestyle lifting task in individuals with and without chronic low back pain | Subjects with recurrent or episodic low back pain |
| Dankaerts (2009) | Discriminating healthy controls and two clinical subgroups of nonspecific chronic low back pain patients using trunk muscle activation and lumbosacral kinematics of postures and movements: a statistical classification model | No CoP variable |
| Ganesh (2015) | Efficacy of the star excursion balance test in detecting reach deficits in subjects with chronic low back pain | No CoP variable |
| Garcez (2021) | Postural adjustments impairments in elderly people with chronic low back pain | No quiet standing condition |
| Gawda (2015) | Evaluation of influence of stretching therapy and ergonomic factors on postural control in patients with chronic non-specific low back pain | No CoP variable |
| Gregory (2008) | Trunk muscle responses to suddenly applied loads: do individuals who develop discomfort during prolonged standing respond differently? | No subjects with chronic low back pain |
| Hamaoui (2002) | Does respiration perturb body balance more in chronic low back pain subjects than in healthy subjects? | No quiet standing condition |
| Hasegawa (2018) | Association of low back load with low back pain during static standing | No CoP variable |
| Hemmati (2018) | Effect of dual tasking on anticipatory and compensatory postural adjustments in response to external perturbations in individuals with nonspecific chronic low back pain: Electromyographic analysis | No CoP variable |
| Hemming (2018) | Non-specific chronic low back pain: differences in spinal kinematics in subgroups during functional tasks | No CoP variable |
| Hodges (1996) | Inefficient muscular stabilization of the lumbar spine associated with low back pain. A motor control evaluation of transversus abdominis | No quiet standing condition |
| Hu (2018) | Using a deep learning network to recognise low back pain in static standing | Some subjects’ ages were older than 65 years |
| Hwang (2019) | Predictors of pain intensity and Oswestry Disability Index in prolonged standing service workers with nonspecific chronic low back pain subclassified as active extension pattern | No group comparison (no healthy control group) |
| Jacobs (2009) | People with chronic low back pain exhibit decreased variability in the timing of their anticipatory postural adjustments | No CoP variable |
| Jacobs (2012) | Low back pain associates with altered activity of the cerebral cortex prior to arm movements that require postural adjustment | No CoP variable |
| Jacobs (2017) | Task-related and person-related variables influence the effect of low back pain on anticipatory postural adjustments | Subjects with recurrent or episodic low back pain |
| Jassi (2017) | Effects of functional taping compared with sham taping and minimal intervention on pain intensity and static postural control for patients with non-specific chronic low back pain: a randomised clinical trial protocol | No group comparison (no healthy control group) |
| Jassi (2021) | Star-Shape Kinesio Taping Is Not Better Than a Minimal Intervention or Sham Kinesio Taping for Pain Intensity and Postural Control in Chronic Low Back Pain: A Randomized Controlled Trial | No group comparison (no healthy control group) |
| Jones (2012) | Individuals with non-specific low back pain use a trunk stiffening strategy to maintain upright posture | No CoP variable |
| Kiers (2015) | Postural sway and integration of proprioceptive signals in subjects with LBP | No CoP variable |
| Koch (2020) | A case control study to investigate differences in motor control between individuals with and without non-specific low back pain during standing | No CoP variable |
| Lauenroth (2019) | Does low back pain affect the reliability of postural regulation? | No group comparison |
| Leitner (2009) | Reliability of posturographic measurements in the assessment of impaired sensorimotor function in chronic low back pain | No CoP variable |
| Li (2014) | Comparison of postural control between healthy subjects and individuals with nonspecific low back pain during exposure to visual stimulus | No quiet standing condition |
| Luoto (1996) | Psychomotor speed and postural control in chronic low back pain patients: A controlled follow-up study | No subjects with chronic low back pain (there is no inclusion criteria for individuals with chronic low back pain |
| Luoto (1998) | One-footed and externally disturbed two-footed postural control in patients with chronic low back pain and healthy control subjects. A controlled study with follow-up | No CoP variable |
| Maribo (2012) | Postural balance in low back pain patients: criterion-related validity of centre of pressure assessed on a portable force platform | No group comparison (no healthy control group) |
| Massé-Alarie (2017) | The side of chronic low back pain matters: evidence from the primary motor cortex excitability and the postural adjustments of multifidi muscles | No CoP variable |
| Matheron (2011) | Vertical heterophoria and postural control in nonspecific chronic low back pain | No group comparison (no healthy control group) |
| Mazaheri (2010) | Postural sway in low back pain: Effects of dual tasks | No CoP variable |
| Mazaheri (2014) | Competing effects of pain and fear of pain on postural control in low back pain? | No CoP variable |
| McCaskey (2018) | Dynamic multi-segmental postural control in patients with chronic non-specific low back pain compared to pain-free controls: A cross-sectional study | No quiet standing condition |
| Mohammadi (2021) | Comparison of the reliance of the postural control system on the visual, vestibular and proprioceptive inputs in chronic low back pain patients and asymptomatic participants | Subjects with recurrent or episodic low back pain |
| Mok (2004) | Hip strategy for balance control in quiet standing is reduced in people with low back pain | Subjects with recurrent or episodic low back pain |
| Mok (2011) | Postural recovery following voluntary arm movement is impaired in people with chronic low back pain | No quiet standing condition |
| Mok (2011) | Changes in lumbar movement in people with low back pain are related to compromised balance | Subjects with recurrent or episodic low back pain |
| Muthukrishnan (2010) | The differential effects of core stabilization exercise regime and conventional physiotherapy regime on postural control parameters during perturbation in patients with movement and control impairment chronic low back pain. | No quiet standing condition |
| Nelson-Wong (2010) | Repeatability of Clinical, Biomechanical, and Motor Control Profiles in People with and without Standing-Induced Low Back Pain | No subjects with chronic low back pain |
| Rhee (2012) | A randomized controlled trial to determine the effect of spinal stabilization exercise intervention based on pain level and standing balance differences in patients with low back pain | No subjects with chronic low back pain |
| Ruhe (2011) | Is there a relationship between pain intensity and postural sway in patients with non-specific low back pain? | No subjects with chronic low back pain |
| Ruhe (2012) | Pain relief is associated with decreasing postural sway in patients with non-specific low back pain | No subjects with chronic low back pain |
| Salavati (2009) | Effect of dual-tasking on postural control in Subjects with nonspecific low back pain | Subjects with recurrent or episodic low back pain |
| Salavati (2009) | Test–retest reliabty of center of pressure measures of postural stability during quiet standing in a group with musculoskeletal disorders consisting of low back pain, anterior cruciate ligament injury and functional ankle instability | Subjects with recurrent or episodic low back pain |
| Schelldorfer (2015) | Low back pain and postural control, effects of task difficulty on centre of pressure and spinal kinematics | No subjects with chronic low back pain |
| Shanbehzadeh (2018) | Attention demands of postural control in non-specific chronic low back pain subjects with low and high pain-related anxiety | Subjects with recurrent or episodic low back pain |
| Sheeran (2019) | Identifying non-specific low back pain clinical subgroups from sitting and standing repositioning posture tasks using a novel Cardiff Dempster-Shafer Theory Classifier | No CoP variable |
| Sherafat (2013) | Intrasession and intersession reliability of postural control in participants with and without nonspecific low back pain using the biodex balance system | No quiet standing condition |
| Sherafat (2014) | Effect of dual-tasking on dynamic postural control in individuals with and without nonspecific low back pain | No quiet standing condition |
| Silfies (2009) | Trunk control during standing reach: A dynamical system analysis of movement strategies in patients with mechanical low back pain | No subjects with chronic low back pain |
| Sipko (2013) | The effect of chronic pain intensity on the stability limits in patients with low back pain | Subjects with recurrent or episodic low back pain |
| Sipko (2013) | Intensity of chronic pain modifies postural control in low back patients | Subjects with recurrent or episodic low back pain |
| Soliman (2017) | The effect of pain severity on postural stability and dynamic limits of stability in chronic low back pain | No CoP variable |
| Sung (2009) | Gender differences in ground reaction force following perturbations in subjects with low back pain | No quiet standing condition |
| Telles (2022) | Concurrent validity of the inertial sensors for assessment of balance control during quiet standing in patients with chronic low back pain and asymptomatic individuals | No English |
| Tsigkanos (2016) | Static and dynamic balance deficiencies in chronic low back pain | No quiet standing condition |
| Tüzün (1999) | Low back pain and posture | No quiet standing condition |
| Van Daele (2010) | Decrease in postural sway and trunk stiffness during cognitive dual-task in nonspecific chronic low back pain patients, performance compared to healthy control subjects | No quiet standing condition |
| Yu (2021) | A Study on the Relationship between Postural Control and Pain-Related Clinical Outcomes in Patients with Chronic Nonspecific Low Back Pain | No quiet standing condition |

##

## Fig. S2. Funnel plots of Duval and Tweedie’s Trim and Fill.


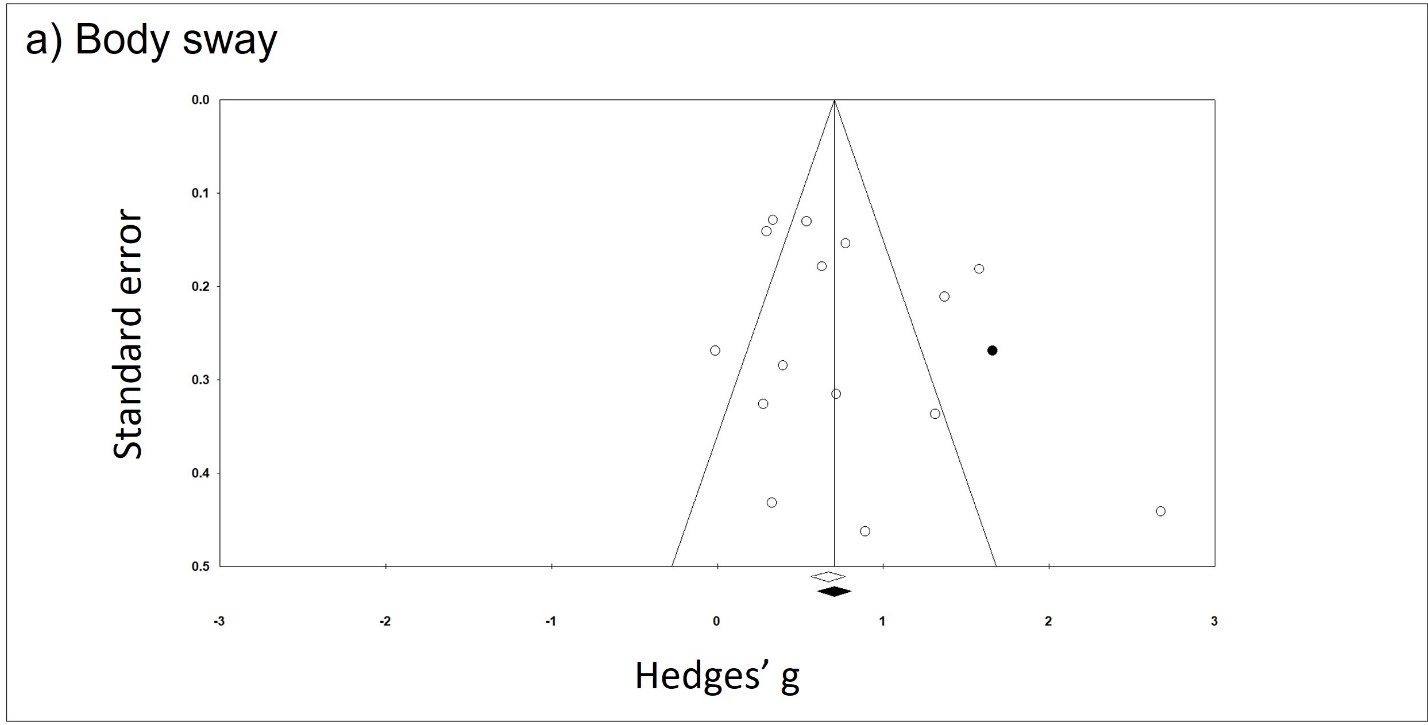

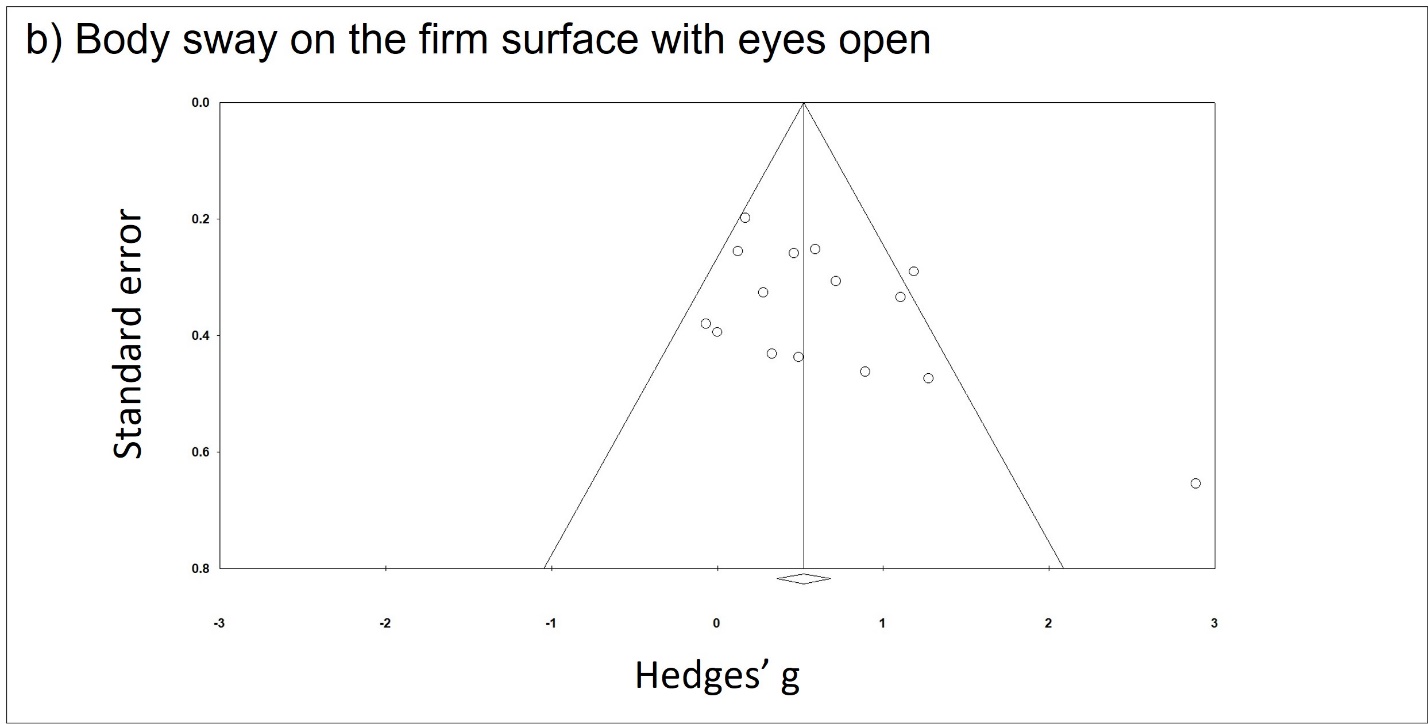

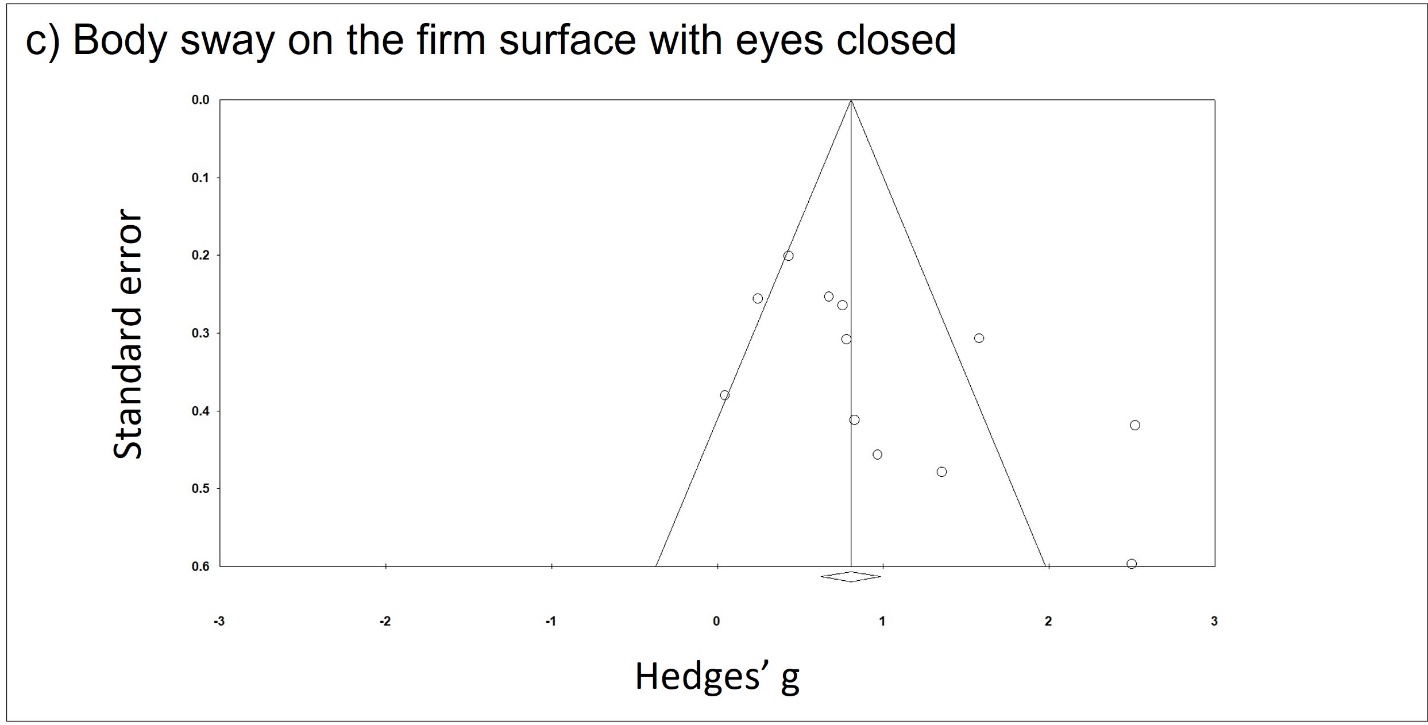

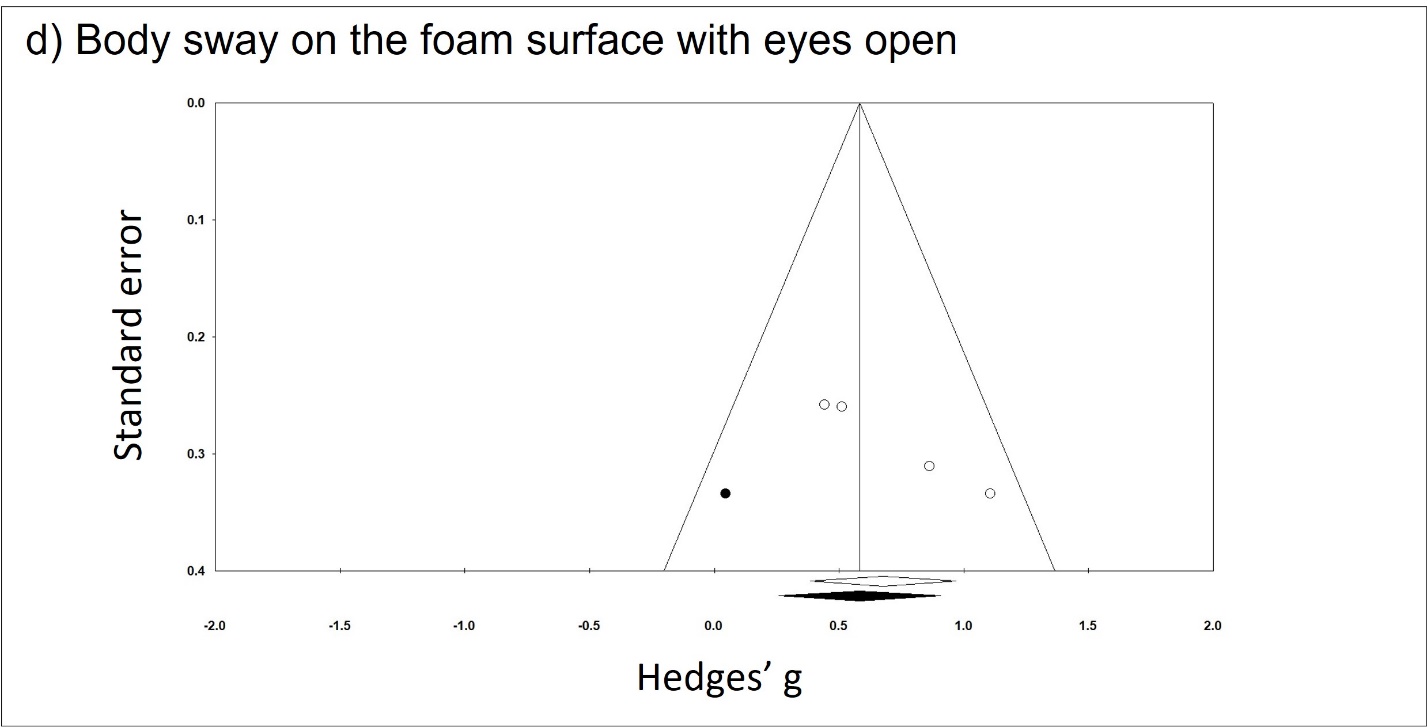

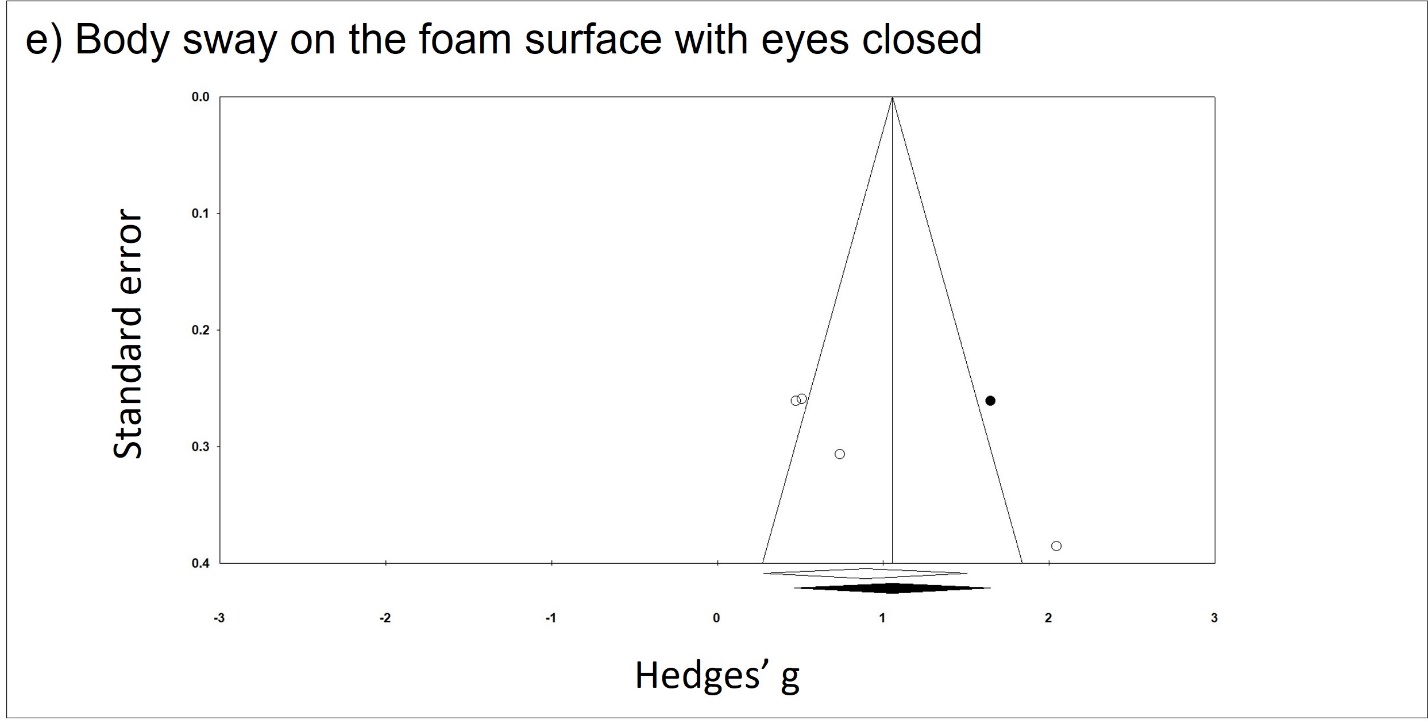

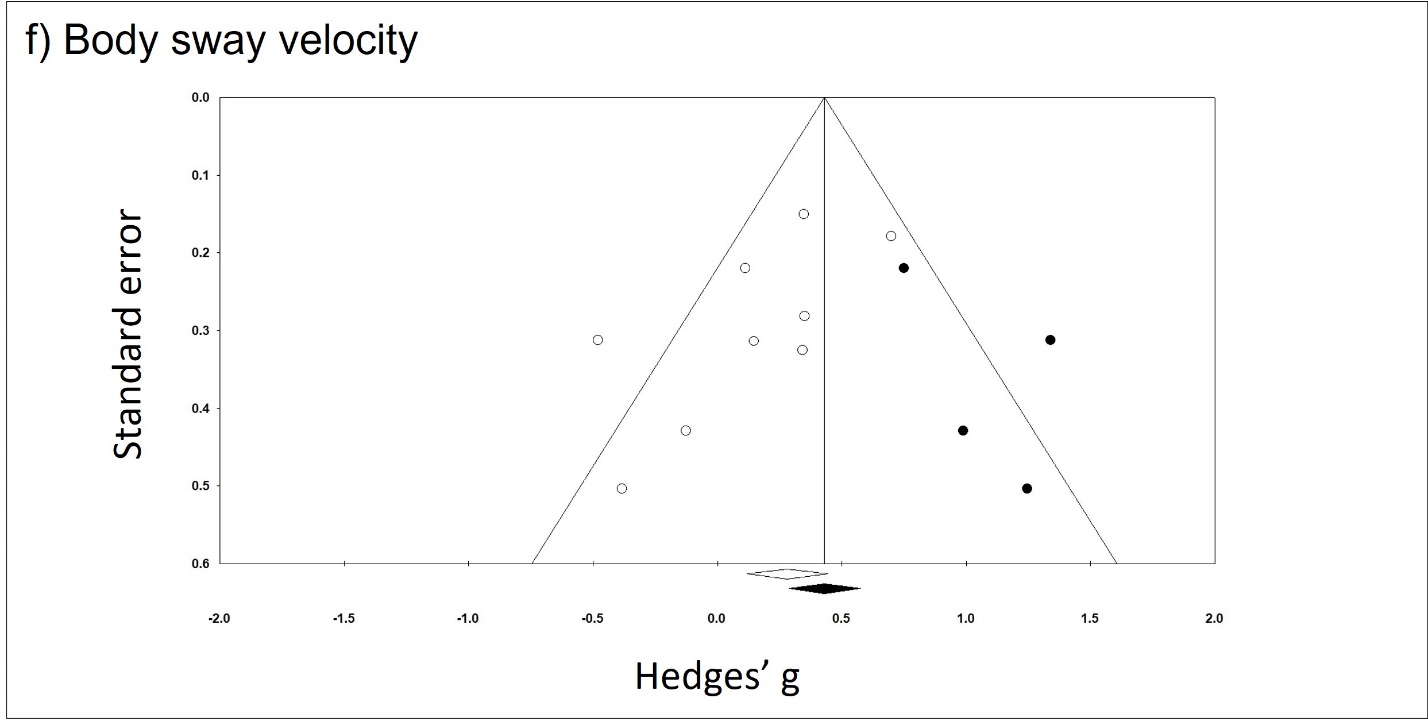

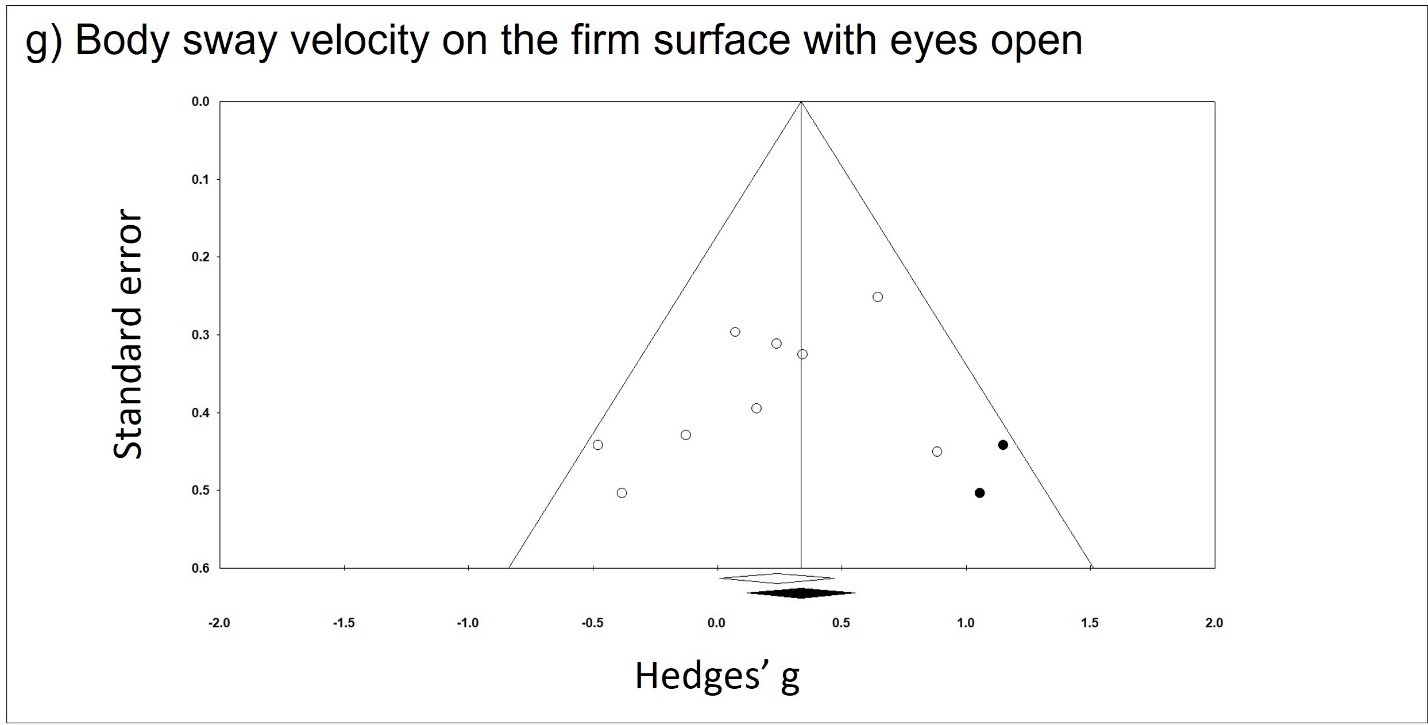

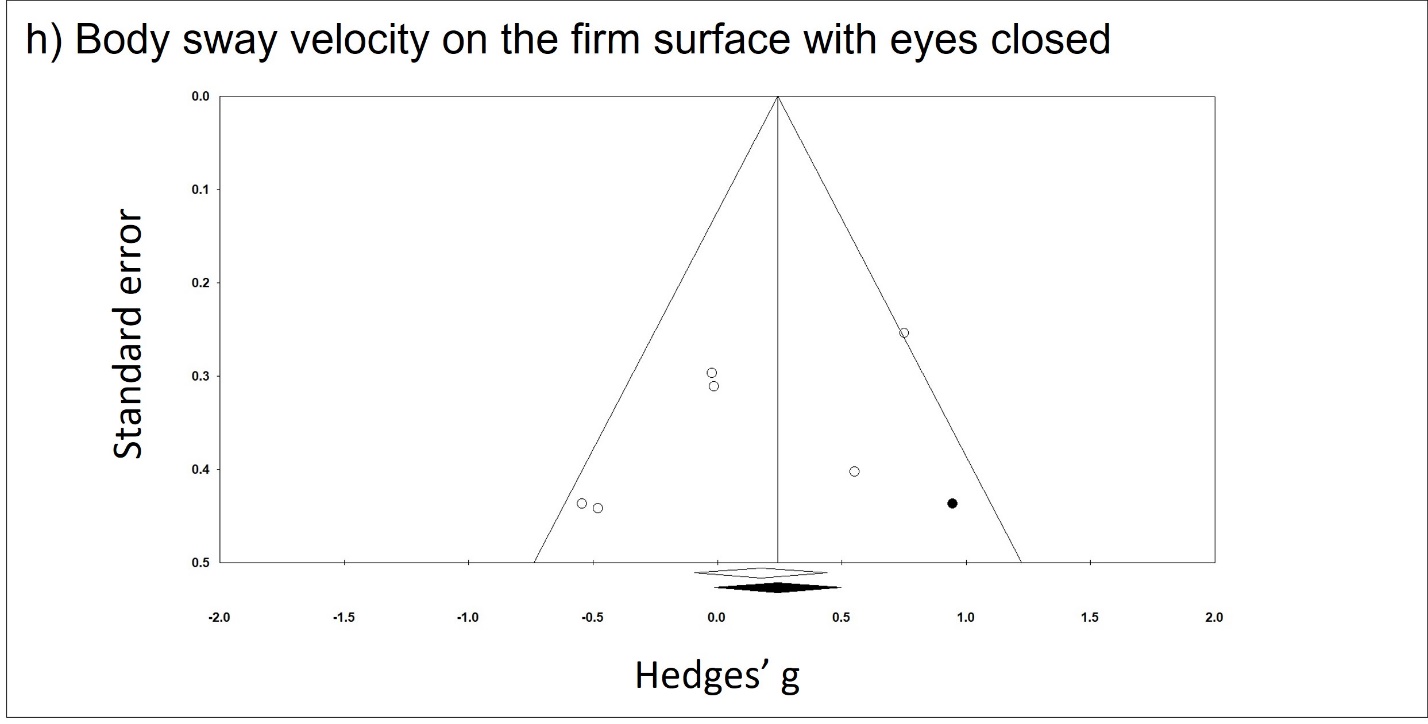


## Table S3. Results of the quality assessment tool for observational cohort and cross-sectional studies.

| Authors | Criteria | | | | | | | | | | | | | | Rating |
| --- | --- | --- | --- | --- | --- | --- | --- | --- | --- | --- | --- | --- | --- | --- | --- |
|  | 1 | 2 | 3 | 4 | 5 | 6 | 7 | 8 | 9 | 10 | 11 | 12 | 13 | 14 |  |
| Abbasi et al. ^42^ | Y | Y | NR | Y | N | Y | N | NA | Y | Y | Y | NR | Y | N | Poor |
| Caffaro et al. ^13^ | Y | Y | NR | Y | Y | N | N | NA | Y | Y | Y | NR | NA | Y | Good |
| Cana-Pino et al. ^14^ | Y | Y | Y | Y | Y | N | N | NA | Y | Y | Y | NR | Y | Y | Good |
| da Silva et al. ^20^ | Y | Y | NR | Y | Y | N | N | NA | Y | Y | Y | NR | NA | Y | Fair |
| della Volpe et al. ^22^ | Y | Y | NR | Y | N | N | N | NA | Y | Y | N | NR | NA | N | Fair |
| Hamaoui & Bouisset ^40^ | Y | Y | NR | Y | N | N | N | NA | Y | Y | Y | NR | NA | N | Poor |
| Lafond et al. ^21^ | Y | Y | NR | Y | N | N | N | NA | Y | Y | Y | NR | NA | Y | Fair |
| Mann et al. ^38^ | Y | Y | NR | Y | N | N | N | NA | Y | Y | Y | NR | NA | N | Poor |
| Nogueira et al. ^6^ | Y | N | NR | Y | Y | N | N | NA | N | N | N | NR | NA | Y | Poor |
| Popa et al. ^22^ | Y | Y | NR | Y | N | N | N | NA | Y | Y | Y | NR | NA | N | Fair |
| Ringheim et al. ^43^ | Y | Y | NR | Y | N | Y | Y | NA | Y | Y | Y | NR | Y | Y | Good |
| Shigaki et al. ^24^ | Y | Y | NR | Y | N | N | N | NA | Y | Y | Y | NR | NA | Y | Fair |
| Sundaram et al. ^44^ | Y | Y | NR | Y | N | N | N | NA | Y | Y | N | NR | NA | Y | Poor |
| Wang et al. ^39^ | Y | Y | NR | Y | N | N | N | NA | Y | Y | Y | Y | NA | Y | Good |
| Yahia et al. ^15^ | N | Y | NR | Y | N | N | N | NA | Y | Y | N | NR | NA | Y | Fair |
| Zhang et al. ^41^ | Y | Y | NR | Y | N | N | N | NA | Y | Y | Y | Y | NA | Y | Good |

*Note*. The quality of studies was assessed using the quality assessment tool for observational cohort and cross-sectional studies designed by National Institute of Health (NIH) ^29^. Criteria 1, Was the research question or objective in this paper clearly stated? Criteria 2, Was the study population clearly specified and defined? Criteria 3, Was the participation rate of eligible persons at least 50%? Criteria 4, Were all the subjects selected or recruited from the same or similar populations or were inclusion and exclusion criteria for being in the study prespecified and applied uniformly to all participants? Criteria 5, Was a sample size justification, power description, or variance and effect estimates provided? Criteria 6, For the analyses in this paper, were the exposure(s) of interest measured prior to the outcome(s) being measured? Criteria 7, Was the timeframe sufficient so that one could reasonably expect to see an association between exposure and outcome if it existed? Criteria 8, For exposures that can vary in amount or level, did the study examine different levels of the exposure as related to the outcome? Criteria 9, Were the exposure measures (independent variables) clearly defined, valid, reliable, and implemented consistently across all study participants? Criteria 10, Was the exposure(s) assessed more than once over time? Criteria 11, Were the outcome measures (dependent variables) clearly defined, valid, reliable, and implemented consistently across all study participants? Criteria 12, Were the outcome assessors blinded to the exposure status of participants? Criteria 13, Was loss to follow-up after baseline 20% or less? Criteria 14, Were key potential confounding variables measured and adjusted statistically for their impact on the relationship between exposure(s) and outcome(s)? All the studies were rated by Good, Fair, or Poor. Y, yes; N, No; CD, cannot determine; NA, not applicable; NR, not reported.

## Table S4. Results of heterogeneity using the Q-test, Tau^2^, I^2^, and prediction interval.

|  | Q-test | |  |  | Prediction interval | |
| --- | --- | --- | --- | --- | --- | --- |
| Meta-analyses | Q-value | DFs | Tau^2^ | I^2^ | Lower limit | Upper limit |
| Body sway | 83.37 | 14 | 0.22 | 83.60 | -0.29 | 1.82 |
| Firm EO | 35.55 | 14 | 0.40 | 60.62 | -0.32 | 1.51 |
| AP | 43.25 | 8 | 0.50 | 81.50 | -0.93 | 2.64 |
| ML | 22.69 | 6 | 0.54 | 73.56 | -1.22 | 1.80 |
| AP-ML | 21.25 | 10 | 0.11 | 52.93 | -0.20 | 1.41 |
| Low pain | 5.97 | 4 | 0.05 | 32.92 | -0.31 | 1.52 |
| High pain | 24.96 | 6 | 0.30 | 75.96 | -0.77 | 2.32 |
| Firm EC | 45.20 | 11 | 0.32 | 75.66 | -0.35 | 2.30 |
| AP | 22.56 | 6 | 0.30 | 73.40 | -0.65 | 2.42 |
| ML | 8.25 | 4 | 0.15 | 51.92 | -0.52 | 1.93 |
| AP-ML | 34.80 | 7 | 0.35 | 79.88 | -0.63 | 2.51 |
| Low pain | 4.85 | 3 | 0.06 | 38.16 | -0.66 | 1.97 |
| High pain | 31.86 | 4 | 0.67 | 87.44 | -1.44 | 4.37 |
| Foam EO | 3.25 | 3 | 0.01 | 7.55 | -0.05 | 1.41 |
| AP | 1.62 | 2 | - | - | - | - |
| ML | 0.33 | 1 | - | - | - | - |
| AP-ML | 1.17 | 2 | - | - | - | - |
| Low pain | 0.75 | 1 | - | - | - | - |
| High pain | 2.48 | 1 | - | - | - | - |
| Foam EC | 13.29 | 3 | 0.30 | 77.42 | -1.82 | 3.61 |
| AP | 4.79 | 2 | 0.10 | 58.29 | -4.72 | 5.56 |
| ML | 0.25 | 1 | - | - | - | - |
| AP-ML | 7.78 | 2 | 0.29 | 74.28 | -7.03 | 9.42 |
| Low pain | 0.44 | 1 | - | - | - | - |
| High pain | 10.96 | 1 | 1.07 | 90.87 | - | - |
| Body sway velocity | 15.08 | 8 | 0.06 | 46.95 | -0.43 | 0.85 |
| Firm EO | 10.02 | 8 | 0.03 | 20.14 | -0.31 | 0.75 |
| AP | 19.05 | 6 | 0.28 | 68.51 | -1.20 | 1.79 |
| ML | 32.01 | 5 | 0.69 | 84.38 | -2.65 | 2.39 |
| AP-ML | 4.48 | 3 | 0.05 | 33.05 | -0.86 | 1.65 |
| Low pain | 7.03 | 3 | 0.16 | 57.34 | -2.03 | 2.16 |
| High pain | 2.72 | 3 | - | - | - | - |
| Firm EC | 11.74 | 5 | 0.16 | 57.40 | -1.15 | 1.35 |
| AP | 5.95 | 3 | 0.11 | 49.54 | -1.50 | 2.02 |
| ML | 13.20 | 2 | 0.60 | 84.85 | -11.66 | 11.69 |
| AP-ML | 8.13 | 3 | 0.17 | 63.10 | -1.92 | 2.26 |
| Low pain | 7.44 | 2 | 0.28 | 73.11 | -7.94 | 8.23 |
| High pain | 0.98 | 1 | - | - | - | - |

*Abbreviation.* DFs: the degree of freedoms; Firm EO: the firm surface with eyes open; Firm EC: the firm surface with eyes closed; Foam EO: the foam surface with eye open; Foam EC: the foam surface with eyes closed; AP, anteroposterior direction; ML, mediolateral direction; AP-ML, sagittal (AP) and horizontal (ML) plane.

## Table S5. Results of publication of biases using Egger’s regression test.

|  | 95% confidence intervals | |  |  |
| --- | --- | --- | --- | --- |
| Meta-analyses | Lower limit | Upper limit | *t*-values | *p*-values (2-tailed) |
| Body sway | -1.60 | 5.31 | 1.16 | .268 |
| Firm EO | -0.14 | 5.50 | 2.05 | .060 |
| Firm EC | 0.39 | 7.75 | 2.46 | .033^*^ |
| Foam EO | 5.35 | 10.94 | 12.54 | .006^**^ |
| Foam EC | 2.25 | 21.18 | 5.32 | .033^*^ |
| Body sway velocity | -4.84 | 0.12 | 2.25 | .059 |
| Firm EO | -6.20 | 1.02 | 1.70 | .134 |
| Firm EC | -11.39 | 2.40 | 1.81 | .144 |

*Note*. The publication biases were assessed using Egger’s regression test. The significant level set to .05. As a result, the significant publication biases were found in the Firm EC, Foam EO, and EC for the body sway variable. To figure the effects of biases, Trim and Fill assessment was used in those analyses (Supplementary Fig. S2).

*Abbreviation.* Firm EO: the firm surface with eyes open; Firm EC: the firm surface with eyes closed; Foam EO: the foam surface with eye open; Foam EC: the foam surface with eyes closed.

^*^*p* < .05; ^**^*p* < .01.
